# Significant modulations of linc001128 and linc0938 with miR-24-3p and miR-30c-5p in Parkinson disease

**DOI:** 10.1038/s41598-022-06539-3

**Published:** 2022-02-16

**Authors:** Maryam Yousefi, Maryam Peymani, Kamran Ghaedi, Shiva Irani, Masoud Etemadifar

**Affiliations:** 1 Department of Biology, Science and Research Branch, Islamic Azad University, Tehran, Iran; 2 Department of Animal Biotechnology, Cell Science Research Center, Royan Institute for Biotechnology, ACECR, Isfahan, Iran; 3 Department of Biology, Faculty of Basic Sciences, Shahrekord Branch, Islamic Azad University, Shahrekord, Iran; 4 Department of Cell and Molecular Biology and Microbiology, Faculty of Biological Science and Technology, University of Isfahan, Isfahan, Iran; 5 Department of Neurology and Isfahan Neurosurgery Research Center, School of Medicine, Isfahan University of Medical Sciences, Isfahan, Iran

**Keywords:** Neuroscience, Genetics, Gene regulation, Long non-coding RNAs, miRNAs

## Abstract

Parkinson disease (PD) is the second most common neurodegenerative disease; the evidence suggests that lncRNAs and miRNAs play an important role in regulating the PD-related genes. The purpose of this research was to introduce two novel lncRNAs as the biomarker of PD diagnosis and treatment. We evaluated the expression profiles of six nodes of two regulatory networks in the PBMCs which had been got from 38 PD patients and 20 healthy individuals by qRT-PCR. Then, we compared the expression of these RNAs in both early and late stages of PD with the controls to determine if their expression could be related to the severity of disease. Further, this study investigated the direct interaction between one of the lncRNAs and target miRNA by using the dual luciferase assay. The results of the expression profiles of six nodes of the two ceRNA networks shown that *linc01128*, *hsa-miR-24-3p* and *hsa-miR-30c-5p* expression were significantly downregulated. While, the *Linc00938, LRRK2* and *ATP13A2* expression were up-regulated in the PBMC of the PD patients, in comparison to the controls. In addition, this study demonstrated that linc00938 directly sponged hsa-miR-30c-5p. The present study, therefore, for the first time, revealed two candidate lncRNAs as the biomarkers in the PD patients.

## Introduction

Parkinson disease (PD) can be regarded as an age-dependent neurodegenerative disorder affecting movement abilities. PD is characterized by progressive loss of dopaminergic neurons in the substantia nigra pars compacta (SNpc)^1^. Degeneration of nondopaminergic neurons usually occurs later in the course of PD^2^. In the industrialized countries, the incidence of PD is estimated to be 8 to 18 per 100,000 people per year. Also, the prevalence of PD in general people, in people over 60 years and in those over the age of 80 years is 0.3%, 1.0% and 3.0%, respectively. The pathology and etiology of PD, which are associated with various risk factors, are largely still unknown. Some of the risk factors contributing to PD include age, gender, environmental factors, and genetic and epigenetic factors^3,4^.

Control of gene expression and protein levels as the major factors in cellular homeostasis are impaired in this disease. Non-coding RNAs (ncRNAs) are risk factors involved in altering the PD-gene expression, playing an important role in PD; although several studies have focused on ncRNAs for the diagnosis and treatment of PD, many have not yet been identified. A class of ncRNAs belongs to microRNAs (miRNAs), which are single-stranded RNAs with a length of almost 21–25 nucleotides. miRNAs are small functional ncRNA classes binding to the 3′-untranslated region (UTR) of the target mRNAs; they are suppressing the target gene expression; they are also involved in various biological processes^5^. Long non-coding RNAs (LncRNAs) are long functional ncRNA classes with more than 200 nucleotides in their length. LncRNAs have multiple modular domains interacting with DNA, proteins and RNA. So, lncRNAs may function by competing with endogenous RNA (ceRNA) for binding the target miRNA, thereby preventing miRNAs from interacting with mRNAs and altering the gene expression; so, they play roles in various biological processes^6^. There are also lncRNAs that have the ability to sponge these microRNAs and alter the expression of miRNAs. Thus, lncRNAs provide novel insights into PD pathogenesis and diagnosis as well as treatment.

Peripheral blood mononuclear cells (PBMCs) are readily available; they are used as a safe source to measure changes in the high quality RNA expression between patients and healthy individuals^7,8^. In this regard, many researches have examined the expression profiles related to PD-candidate miRNAs, lncRNAs and mRNAs in PBMCs^9,10^.

The gene expression of leucine-rich repeat kinase 2 (*LRRK2*) and ATPase type 13A2 (*ATP13A2*) was related to the PD disease, although *miR-30c* and *miR-24* could alter the expression of target genes and were associated with PD. There are also lncRNAs that have the ability to sponge these microRNAs and alter the function of miRNAs^11,12^. The aim of this study was not only to demonstrate the expression profiles related to the set of candidate mRNAs, miRNAs and lncRNAs, but also to present lncRNAs as some new biomarker for the PD pathogenesis, diagnosis and treatment. As well, in this study, to show the direct interaction between lncRNA and target miRNA, as well as its ability to sponge microRNA, transfection of them to the cell was done and the dual luciferase method was used.

## Methods

### Bioinformatics study

In this work, two bioinformatics studies were performed. The first study was carried out to identify the candidate miRNAs, lncRNAs and target mRNAs that were related to PD, and to construct a triple network between candidate mRNAs, miRNAs and lncRNAs (ceRNA). Therefore, three steps were required to select the candidate coding and non-coding RNAs. The first step was to candidate miRNAs, For this step, miRNAs that had been investigated through literature and miRNAs were examined via strong validation, using *MiRTarBase* database (*v.8.0*) (125 miRNAs) were selected^13^. The second one was to candidate lncRNAs; in this step, the names of the mentioned miRNAs in the *DIANA-LncBase Experimental* )*v.2*( database were used to obtain the target lncRNAs (387 lncRNAs)^14^. Screening was then performed to identify the final lncRNAs candidate. Therefore, from the listed lncRNAs, those expressed in both blood and brain were selected using the *DIANA-LncBase experimental* database (159 lncRNAs), Then, lncRNAs identified in the literature as being related to PD, as well as those associated with less than two miRNAs, were removed (66 lncRNAs). The third step was to find the genes corresponding to the target miRNAs of LncRNAs; the important genes were selected through literature review and key genes in the PD pathway in the *KEGG* database^15^ and *DisGeNET* (*v.7.0*)^16^ (PARK1-PARK22). So, LncRNAs interacting with important PD genes were selected by target miRNAs (34 lncRNAs). Then, the association of lncRNAs with other neurodegenerative diseases, as reported by the previous studies, such as Alzheimer's disease, were ignored; these could be considered specific for PD (28 lncRNAs). Finally, those lncRNAs and mRNAs whose distance was less than 50 kb between producing genes locus were selected (2 lncRNAs, 2 mRNA); finally, the target miRNAs of the two final lncRNAs were selected.

Several bioinformatics tools were employed in this work. The association between lncRNAs and Parkinson disease was obtained from the *LncRNADisease* (*v.2.0*) database and literature review^17^. The sequence of the selected lncRNA transcripts and location of their expression were determined using *LNCipedia* (v.5.2)^18^ and *NONCODE* (v.5.0) databases, respectively^19^. The interaction of miRNAs and target mRNAs was obtained from the *miRDB* database^20^. Also, the potential interaction of miRNAs and target lncRNAs was predicted using the *DIANA-LncBase Experimental* database. The interaction of lncRNAs and target mRNAs was obtained from the *LncRRIsearch* database^21^.

As well, the miRNA-based network which included *hsa-miR-30c-5p*, *hsa-miR-24-3p* and their PD-related target genes was constructed using the *Cytoscape* (*v3.7.2*) software^22^.

Regarding the section titled “Statistical analysis and selection of miRNA and target lncRNA for the dual luciferase assay”, a second bioinformatics study was required to identify the sequences of the selected lncRNA and the precursor miRNA to construct the recombinant plasmid for the dual luciferase assay.

The sequences of all *linc00938* transcripts were obtained from the *LNCipedia* database; as well, the selected miR-30c sequence was obtained from the *miRBase* (*v.22.1*) database^23^. Then, we input the lncRNA and miRNA sequences into the *RNA22* (*v2*) database to predict the miRNA-lncRNA binding site^24^. On the other hand, the sequence of the genes generating miR-30c was obtained using the *NCBI* database^25^ for the PCR amplification. After that, we predicted the binding sequence of miR-30c in the *linc00938* wild type and mutant by using *RNAhybrid-BiBiServe2*^26^.

### Samples collection

Age and gender are important risk factors for PD^27^. So, in the present research, to deeply investigate the effect of changes in the coding and non-coding RNA expression in this disease, sampling was performed in the patients and healthy groups who had no special difference in terms of age and sex.

After the informed consent was signed by the participants, the collection of blood samples was done from 38 Parkinson's patients who had referred to the Neurology Clinic. The confirmation of the diagnosis of Parkinson’s in each of these patients was done by referring a certified neurologist in the field of movement disorder, based on the UK PD Society Brain Bank criteria^28^. The clinical severity and stage of PD symptoms of each patient were evaluated by applying the Unified Parkinson's Disease Rating Scale (UPDRS), based on which the stage of the disease was determined^29^; therefore, ten patients with the slight state (stage 1), seventeen patients with the mild state (stage 2), ten patients with the moderate state (stage 3) and one patient with the severe state (stage 4) participated in this study. The Montreal Cognitive Assessment (MoCA) was also completed and scored to determine the patients' cognitive impairment^30^. Patients suffering from other neurological diseases, unusual Parkinsonism, drug-induced secondary parkinsonism, diabetes, renal impairment, infection, tumor, cognitive impairment (Montreal score less than 26), and cardiovascular and cerebrovascular diseases were not included in the study. Blood samples were also taken from 20 volunteers as a healthy group who had no personal or family history of neurological and psychiatric diseases; also, the age and lack of other diseases mentioned were matched with the patients.

### Isolation of PBMCs

Four mL of the all participants' whole blood was got in some EDTA-containing (anti-coagulant) tubes. The blood samples were diluted with the same amount of Phosphate Buffered saline (PBS¯) (Gibco, Thermo Fisher Scientific, USA); then the isolation of peripheral blood mononuclear cells (PBMCs) from whole blood was done using Lymphodex separating solution density gradient centrifugation (Inno-Train Diagnostik GmbH, Germany), based on the manufacturer’s protocol. Finally, the washing of the cell precipitate was done twice with PBS^¯^.

### RNA extraction

Total RNAs, including mRNAs, lncRNAs and miRNAs, were extracted from PBMCs, according to the manufacturers’ instructions by applying the TRIzol reagent (Invitrogen, Carlsbad, USA). This included Phase Separation, RNA Precipitation, RNA Wash and Determination of the RNA Concentration by employing a NanoDrop 2000/2000c spectrophotometer (Thermo Fisher Scientific, USA).

### CDNA synthesis

In this study, two kits were used for the cDNA synthesis; one of them was related to the cDNA synthesis of mRNAs and lncRNAs (Yektatajhiz cDNA synthesis kit, Iran); BONmiR kit (Bonbiotech, BONmiR, Iran) was used for the cDNA synthesis of miRNAs. As the first step in the cDNA synthesis of lncRNAs and mRNAs, the treatment of all RNA samples was done with *DNase I* and *RNase inhibitor* (Fermentas, Thermo Fischer Scientific, USA) to eliminate possible genomic DNA (gDNA) contamination. Then, according to the protocol, random hexamer (RH), *RNA inhibitor (RI*), dNTP and *M-MLV* were added to the microtubes containing the treated RNAs (500 ng) for the cDNA synthesis.

As well, the first step in miRNAs cDNA synthesis was polyadenylation of miRNAs; then, BON-RT adaptor (10 μM), *Reverse Transcriptase (RT)* and dNTP mix (100 mM) were added to RNA-containing microtubules (2500 ng) for the cDNA synthesis.

### Statistical analysis and selection of miRNA and target lncRNA for the dual luciferase assay

All qRT-PCR experiments were performed in triplicate. Data were analyzed using *GraphPad Prism 8.0* (GraphPad Software, USA); data were expressed as the means ± standard error of the mean (SEM). The D'Agostino and Pearson test was then used to determine the normal distribution for each variable. The unpaired non-parametric Mann–Whitney and the parametric t-test were then applied for the evaluation of the significance of differences between the two groups; as well, the unpaired non-parametric Kruskal–Wallis and parametric Ordinary one-way ANOVA for n groups were used. A probability value less than 0.05 (p < 0.05) was assumed as a statistically significant difference. Also, the receiver operating characteristic (ROC) curve analysis was conducted for each RNA.

The statistical analysis of the qRT-PCR experimental data was done to choose the target lncRNA and the related miRNA with a higher probability of interaction for the dual luciferase assay.

### Plasmid construction

Based on the database prediction, we selected about 140 bp of the *linc00938* sequence that had the *miR-30c* binding site. Therefore, the wild-type, *linc00938,* including the seed region of *hsa-miR-30c* and the mutant-type *linc00938,* which was altered at the *hsa-miR-30c* binding site, were purchased. Also, we investigated the interactions between MT and miRNA, as well as those between WT and miRNA, using *RNAhybrid-BiBiServe2*. Then, the mutant and wild-type of *linc00938* were double digested with *XhoI* and *NotI* enzymes (Thermo Fisher Scientific, USA) and gel extracted (QIAquick Gel Extraction Kit, Germany); they were cloned in-frame between the same restriction sites in the luciferase reporter psiCHECK-2. PsiCHECK-2 vector contained the ampicillin (Amp) resistance gene. The recombinant plasmids were obtained through transformation of Escherichia coli (E. coli) DH5a bacteria as a host. Then the Amp-resistant colonies were selected and confirmed by colony-PCR using psiCHECK-2 forward primer, 5′-AGAAGTTCCCTAACACCGAGT-3′ and psiCHECK-2 reverse primer, 5′-GCGTCAGACAAACCCTAACCAC-3′.

On the other hand, in chromosome 6, GRCh38.p13 region can produce the precursor miR-30c-5p (miR-30c-2); so, DNA was extracted from whole blood using the salting out method. The length of the precursor miR-30c-5p (pre-miRNA), which was about 300 bp, was amplified by PCR using the forward primer, 5′-AAGCTTAGAGCCATAATTTAGTCCCAA-3′ (HindIII restriction site underlined) and the reverse primer, 5′-GGATCCCTCAGAAACAAACACGGGAT-3′ (BamHI restriction site underlined). Then, it was extracted from the gel and first cloned into the pTZ57R/T vector; the recombinant plasmids were obtained through transformation in E. coli DH5a bacteria as a host. The Amp-resistant colonies were then selected and confirmed by colony-PCR using M13 forward (vector primer), 5′-GCCAGGGTTTTCCCAGTCACGA-3′ and M13 reverse, 5′-GAGCGGATAACAATTTCACACAGG-3′. Recombinant pTZ57R/T vector was digested with *HindIII* and *BamHI*, gel extracted and cloned in-frame between the same restriction sites in the pBud-EGFP plasmid. pBud-EGFP is Bleocin (Zeocin) resistance^31^; so, Zeocin-resistant colonies were selected and confirmed by colony-PCR using the CMV forward (vector forward), 5′-CGCCATGTTGACATTGATTATTG-3′ and miR-30c reverse primer.

### Cell culture

HEK293T (human embryonic kidney 293 T) cells were purchased from Pasteur Institute, Iran. The culturing of the cells was done in the Dulbecco's modified Eagle's medium (DMEM) (Gibco, USA) which had been supplemented with 10% fetal bovine serum (FBS, Gibco, USA), 1% mM l-glutamine (Gibco, USA) and 1% penicillin/streptomycin (100 µg/ml, Gibco, USA). The mentioned cells were kept in the humidified incubator to the temperature of 37 °C with 5% CO2.

### Plasmids transfection

The cells were seeded in some 12-well plates with the density of 1.4 × 10^5^ cells per well. Regarding the instructions of Lipofectamine, 2000 transfection reagent (Invitrogen, Carlsbad, CA, USA), transfection of the plasmids to the cells was performed when cell confluence ranged from 70 to 90%. The cultured cells were divided into four groups: psiCHECK (empty vector) + pBud-mir-30c (as the negative control or NC), psiCHECK-linc00938 (WT) + pBud-mir-30c, psiCHECK-linc00938 (MT) + pBud-mir-30c, and cells without any vector (as the Blank). After transfection for 48 h, before the luciferase assay and qRT-PCR were performed, the transfection of pBud-mir-30c into the cells was confirmed using a fluorescent microscope (Olympus Co, Japan); fluorescence images were captured by an Olympus DP70 camera.

### Dual luciferase reporter assay

In this study, the dual luciferase activity was performed to demonstrate the direct interaction between *miR-30c* and *linc00938*. The psiCHECK-2 vector containing the *Renilla* luciferase was applied as the primary reporter gene; also, the *firefly* luciferase served as the second reporter gene; the *firefly* luciferase allowed the *Renilla* luciferase expression normalization. The sequences of lncRNAs were inserted into the *Renilla* luciferase 3′ UTR in the psiCHECK-2 vector. Comparison of the ratio of *firefly* luciferase activity*/Renilla* luciferase activity was done in the cells which had been transfected with miR-30c and linc00938 (WT, MT, control), to normalize the luciferase activity.

### QRT-PCR

At first quantitative real-time PCR (qRT-PCR) was performed to identify the differential expression (DE) of set of candidate mRNAs, miRNAs and lncRNAs. So, the expression level of *LRRK2, ATP13A2* mRNAs, *linc00938, linc01128* lncRNAs and hsa-*miR-30c-5p*, and *hsa-miR-24-3p* miRNAs were analyzed by applying the SYBR Green Master Mix (TaKaRa, Japan) with Step One Plus Real-Time PCR thermal cycler (Applied Biosystems, USA). *U6, GAPDH* and *18 s* were used for normalization. The miRNAs primer sets (Catalog #BON209002, Bonbiotech, BONmiR, Iran) were purchased for the qRT-PCR. However, the mRNAs as well as lncRNAs specific Primers were designed by using the *Beacon* designer (version *8.2*, Palo Alto, CA, USA) and *Oligo7* software (Molecular Biology Insight, CO, USA), whose sequences are shown in Table 1.

**Table 1:** Sequences of designed primers.

Name	Sequences	
*LRRK2*	Forward	5′-TCGTTGGCACACATTTGGAT-3′
	reverse	5′-GGAACCCTCGCTTATTCAGG-3′
*ATP13A2*	Forward	5′-CAGCTCCTCAGTTTCATCCGT-3′
	reverse	5′-TCCCAGCCATCATCCAGACCAC-3′
*LINC01128*	Forward	5′-ATTATTGCTCTTGGACGACCC-3′
	reverse	5′-CTCCGGCAACTTTCATGACT-3′
*LINC00938*	Forward	5′-AATGCCAACGACTTCTACCAC-3′
	reverse	5′-GTCACCTCAGCTTTCCGTTC-3′
*GAPDH*	Forward	5′-CCACTCCTCCACCTTTGACG-3′
	reverse	5′-CCACCACCCTGTTGCTGTAG-3′
*18s*	Forward	5′-CGGACACGGACAGGATTG-3′
	reverse	5′-TCGCTCCACCAACTAAGAAC-3′

A total of 45 cycles were run for miRNAs qRT-PCR; the polymerase chain reaction was carried out in two main steps; this included holding the reagents at the temperature of 95 °C for 5 s (denaturation); this was followed by the temperature of 60 °C for a period of 30 s (annealing and extension). As well, a total of 40 cycles were run for mRNAs and lncRNAs qRT-PCR; the polymerase chain reaction was carried out in three main steps; this included holding the reagents at the temperature of 95 °C for a period 5 s (denaturation); this was followed by the temperature gradient for 10 s (58 °C for *LRRK2, LINC01128, LINC00938 and 18 s.* 60 °C for *GAPDH.* 62 °C for *ATP13A2*) (annealing); finally, the reagents were kept at 72 °C for 30 s (extension). The relative expression levels were estimated by applying the comparative C_T_ method.

Then, qRT-PCR was carried out to confirm the production of the mature miR-30c in the transfected cells. After transfection of pBud-mir-30c in HEK293T for 48 h, the cells were released using the TRIzol reagent; then RNA extraction and qRT-PCR were performed according to the previous section, titled “RNA extraction”. Also, qRT-PCR was performed to confirm the expression of LRRK2 in various transfected cells (psiCHECK (empty vector) + pBud-mir-30c (NC), psiCHECK-linc00938 (WT) + pBud-mir-30c, psiCHECK-linc00938 (MT) + pBud-mir-30c, and cells without any vector (Blank)) after transfection for 48 h.

All methods were carried out in accordance with relevant guidelines and regulations.

### Ethics approval

The approval of this study (IR.ACECR.ROYAN.REC.1400.045) was obtained from the local ethics committee of Royan Institute.

## Results

### Selection of mRNAs, lncRNAs and miRNAs

The bioinformatics study was performed to identify candidate miRNAs, lncRNAs and target mRNAs that were related to PD, and to construct a regulatory triple network between candidate mRNAs, miRNAs and lncRNAs (ceRNA). Therefore, three steps were required to select from candidate coding and non-coding RNAs. As detailed in the methods section, as the first step, hsa-*miR-30c-5p* and the *hsa-miR-24-3p* miRNAs were selected from 125 miRNAs. In the second one, *linc00938* and *linc01128* lncRNAs were selected from 387 lncRNAs; as the third step, *LRRK2, ATP13A2* mRNAs were selected. In other words, we considered *hsa-miR-24-3p*, *linc01128* and *ATP13A2* as the first network and hsa-*miR-30c-5p*, *linc00938* and *LRRK2* as the second one.

The *DIANA-LncBase Experimental* (*v.2*) database shows the interaction score of miRNAs and lncRNAs using the immunoprecipitation assay; these two lncRNAs, as examined in this study, had scores above 0.7 out of 1.00 for the interaction with miRNAs (scores signified the interaction strength).

### Samples characteristics

Table 2 describes the demographic as well as clinical characteristics of the PD patients and controls. Statistical analysis revealed no significant correlation in regard to age, gender and BMI between PD patients and controls groups; however, significant differences were found in terms of Disease Duration between the two stages of PD (early stage and late stage). On the other hand, the disease duration was found to be much higher in the late stage PD patients, as compared to the early one.

**Table 2:** Demographic and clinical details of PD patients and controls. p-value was calculated using Unpaired t test.

Demographics	Control	PD	Early stage UPDRS score (%)		Late stage UPDRS score (%)		P-value
			> 25 (Stage I)	25–50 (Stage II)	51–75 (Stage III)	> 75 (Stage IV)	
Sample size	20	38	10	17	10	1	-
Gender							
Male (%)	10 (50%)	21 (55%)	6	9	5	1	0.6468
Female (%)	10 (50%)	17 (45%)	4	8	5	0	
Age range (year) Mean ± SD	59.45 ± 8.15	64.45 ± 10.96	65.11 ± 10.40		62.82 ± 12.59		0.078
Disease Duration (month) Mean ± SD	-	59.90 ± 54.39	43.85 ± 31.69		99.27 ± 77.04		0.0030*
BMI Mean ± SD	27.57 ± 5.55	25.86 ± 4.22	26.05 ± 4.60		25.38 ± 3.24		0.1929

*The difference between the two groups was significant (P > 0.05).

### Downregulation of *Linc01128* and *hsa-miR-24-3p* in the PBMC of PD patients

**Figure 1:**
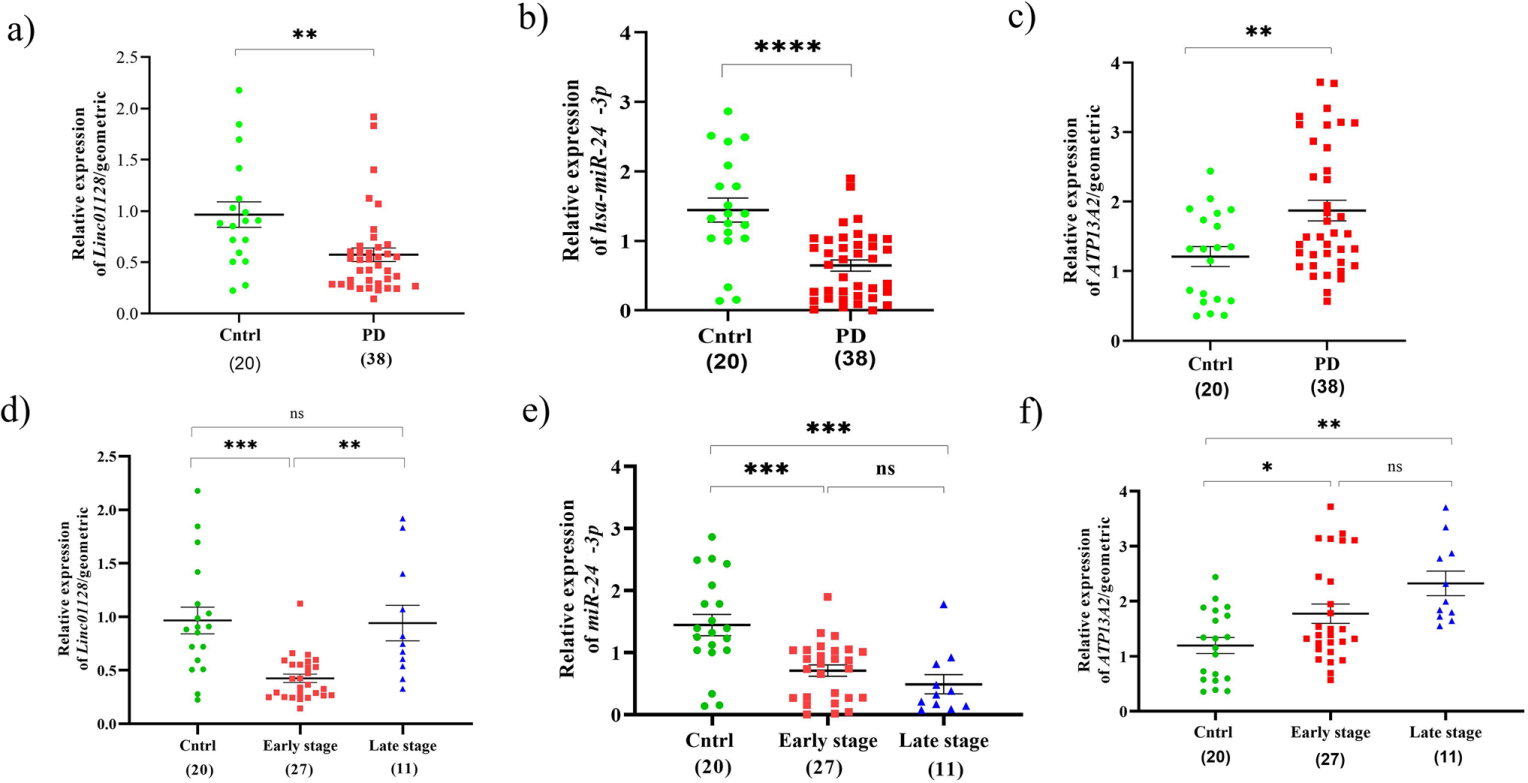
Evaluation of the lncRNA, miRNA and mRNA relative expression as formed in the first regulatory network. QRT-PCR analysis of RNA expression in the PBMC of: (**a**–**c**) control subjects and PD patients. (**d**–**f**) control subjects and PD early and late stage groups, based on the UPDRS score. All data are represented as mean ± SEM (***p ≤ 0.001, **p ≤ 0.01, *p ≤ 0.05, and ns p > 0.05).

**Figure 2:**
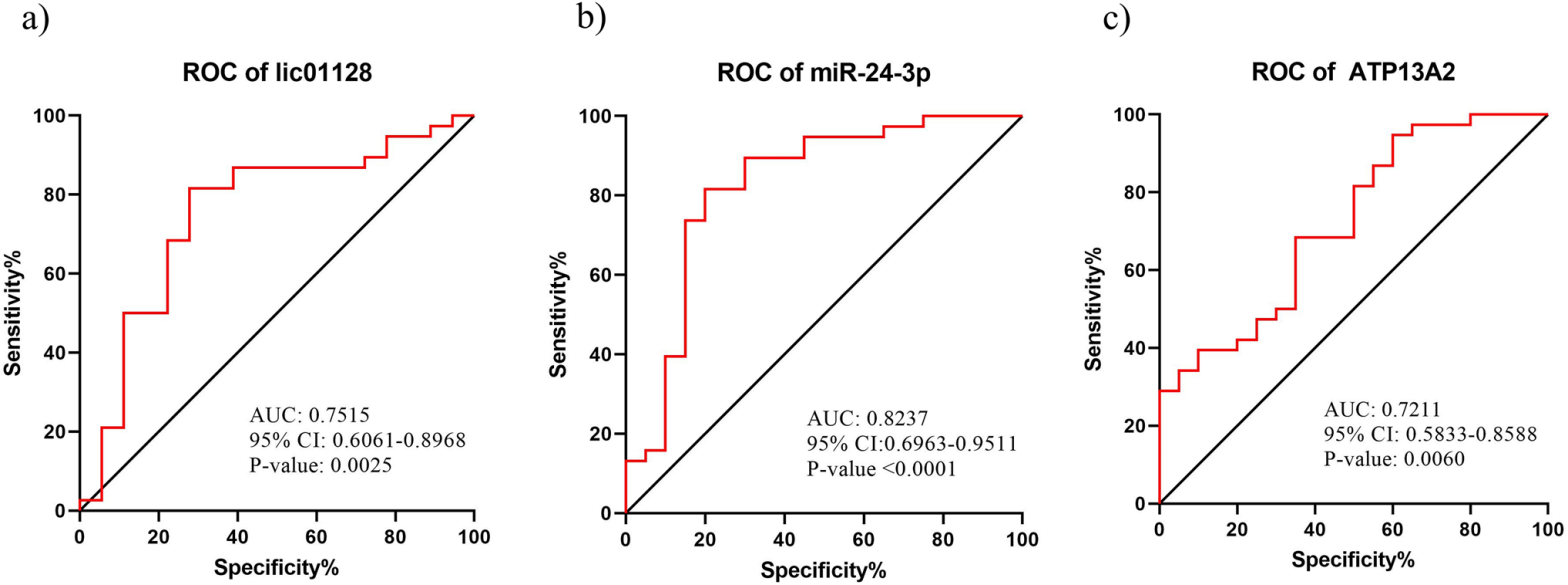
Analysis of the ROC curve for: (**a**–**c**) linc01128, miR-24-3p and ATP13A2 in the PD patients, as compared to the healthy controls.

Statistical analysis of the qRT-PCR experimental data of the first network revealed that lncRNA *linc01128* and *hsa-miR-24-3p* were significantly downregulated, while mRNA *ATP13A2* was upregulated in the PD patients PBMC, in comparison to the healthy samples (Fig. 1a–c). Moreover, we analyzed the expression of RNAs on both early and late stages related to the PD patients (according to on the UPDRS scores), in comparison to the controls. The results of *linc01128* expression demonstrated that *linc01128* was considerably reduced in the patients who were in the early stage, as compared to the healthy and advance ones (Fig. 1d). On the other hand, *hsa-miR-24-3p* showed significant reduction in both early and late stage patients, in comparison to the healthy individuals (Fig. 1e). Meanwhile, *ATP13A2* was significantly overexpressed in both early and late stage patients, in comparison to the healthy individuals (Fig. 1f). Also, ROC curve analysis, defined as sensitivity plotted against specificity, was performed to assess the performance of the significantly differential expression of *linc01128*, *miR-24-3p* and *ATP13A2* to discriminate the PD patients from the healthy subjects (Fig. 2a–c). In this regard, range of areas (AUC), under ROC curves for *linc01128, miR-24-3p* and *ATP13A2*, was 0.7515, 0.8237 and 0.7211, respectively. Thus, the results of such ROC analyses indicated a fair distinction between the PD and control groups.

### Upregulation of *Linc00938* and downregulation of hsa-miR-30c-5p in the PBMC of PD patients

**Figure 3:**
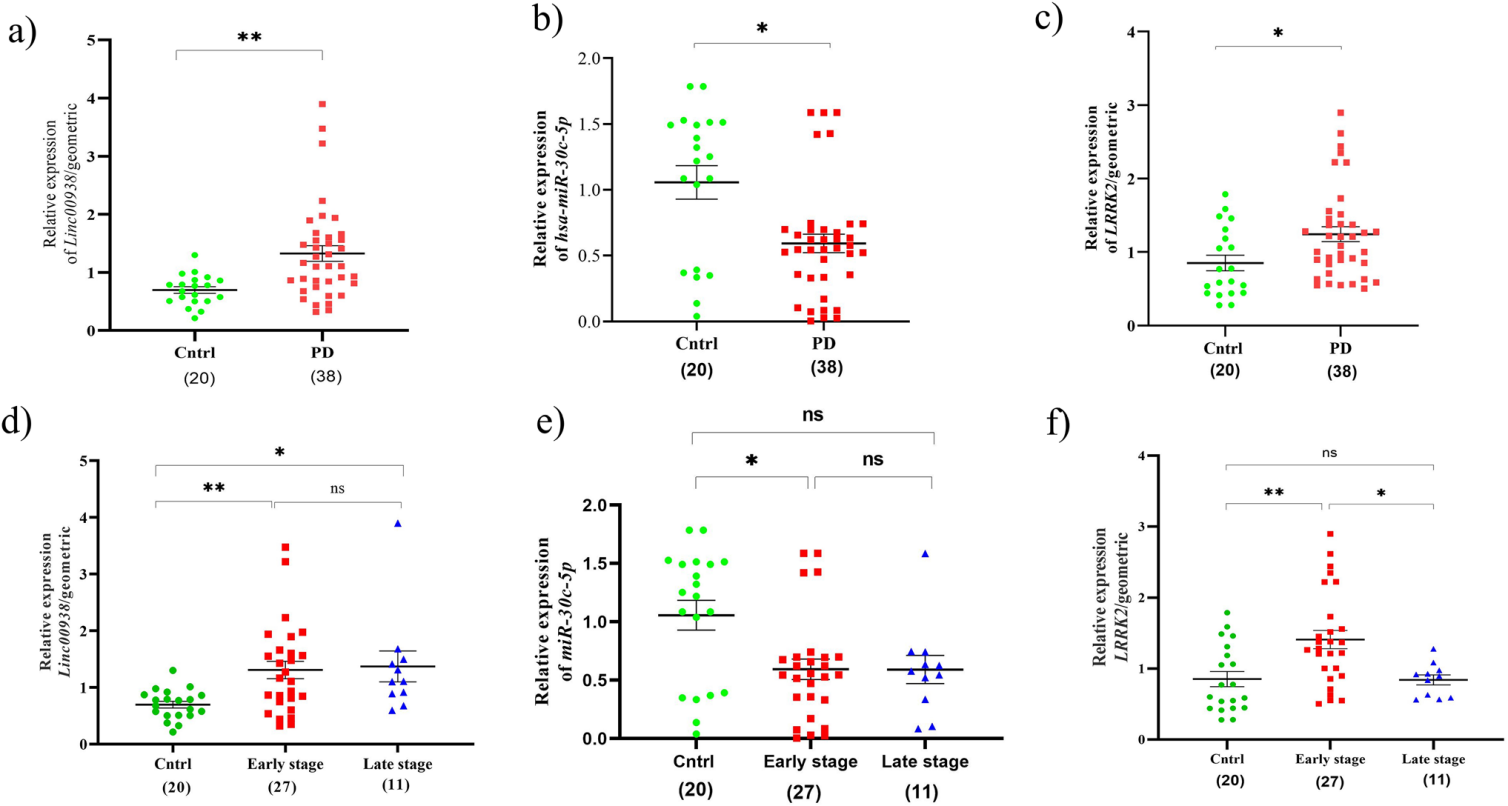
Assessment of the relative RNA expression related to lncRNA, miRNA and mRNA as formed in the second regulatory network. QRT-PCR analysis of the RNA expression in the PBMC of: (**a**–**c**) control subjects and PD patients. (**d**–**f**) control subjects and PD early and late stage groups, based on the UPDRS score. All data are represented as mean ± SEM (**p ≤ 0.01, *p ≤ 0.05, and ns p > 0.05).

**Figure 4:**
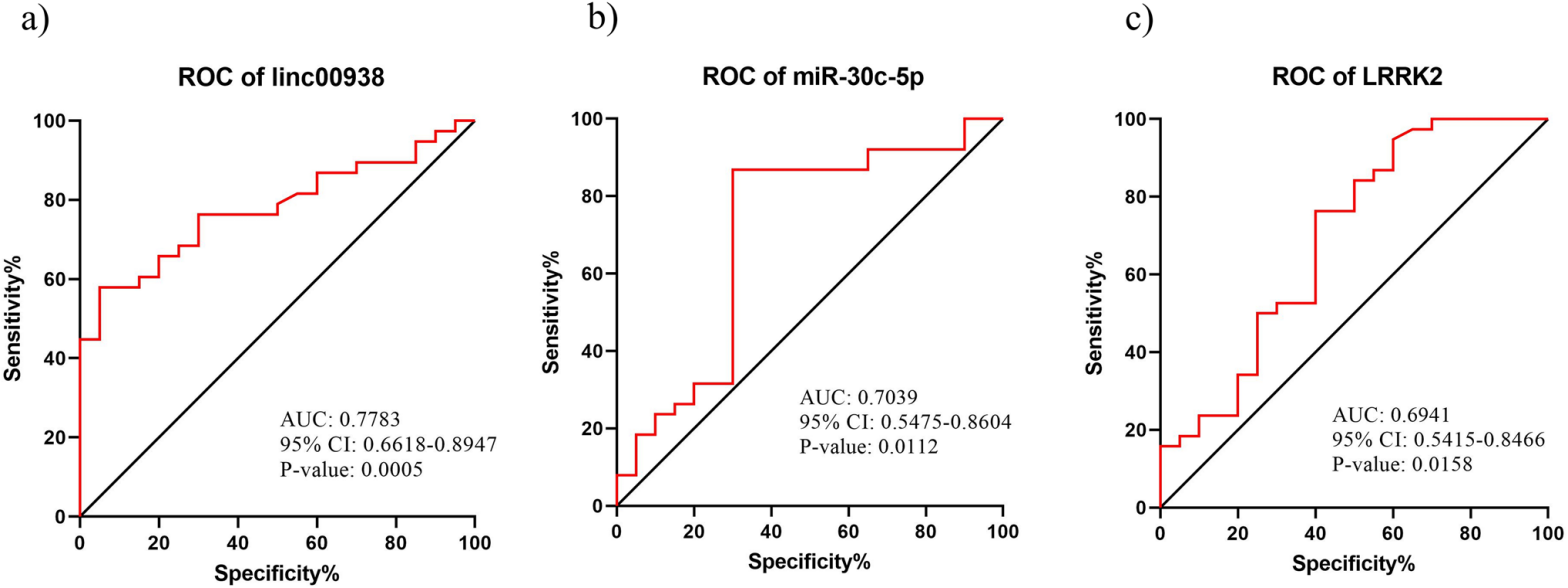
Analysis of the ROC curve for: (**a**–**c**) linc00938, miR-30c-5p and LRRK2 in the PD patients, as compared to the healthy controls.

The results of the second network RNAs expression comparison in the PBMC of the PD patients to the controls, showed that *Linc00938* was significantly increased; meanwhile, *hsa-miR-30c-5p* was significantly reduced; on the other hand, the *LRRK2* expression was significantly overexpressed (Fig. 3a–c). Also, we analyzed the expression of these RNAs on the PD patients´ stages, as compared to the controls. *Linc00938* was considerably increased in both early and late stage patients, in comparison to the healthy individuals (Fig. 3d). However, *hsa-miR-30c-5p* was significantly reduced in the early stage, in comparison to the controls (Fig. 3e); meanwhile, *LRRK2* was remarkably overexpressed in the early stage patients, as compared to control and advanced stage groups (Fig. 3f). Also, ROC curve analysis was performed to assess the performance of the significantly differential expression of *linc00938*, *miR-30c-5p* and *LRRK2* to discriminate the PD patients from the healthy groups (Fig. 4a–c). In this regard, range of areas (AUC), under ROC curves for *linc00938*, *miR-30c-5p* and *LRRK2*, was 0.7783, 0.7039 and 0.6941, respectively. Thus, the results of these ROC analyses indicated a fair distinction between the PD and control groups.

In this regard, the name of the analysis test, mean difference (95% CI) and P-value for each of the examined RNAs within each group are represented in Table 3. On the other hand, based on the results, there was the possibility that *linc00938* and *hsa-miR-30c-5p* could have a higher probability of direct interaction with each other; so, we used the dual luciferase assay to find the direct interaction of *Linc00938* and *hsa-miR-30c-5p.*

**Table 3:** Results of the RNA expression analysis.

Multiple comparisons	RNAs name	Name of analysis test	Mean difference (95% CI)	P-value
Controls vs. PD	Hsa-miR-24a-3p	Unpaired t test	− 0.7990^a^ (− 1.131 to − 0.4674)	< 0.0001*
	Hsa-miR-30c-5p	Mann–Whitney test	− 0.6839^b^ (− 0.8702 to − 0.1985)	0.0105*
	Linc01128	Mann–Whitney test	− 0.3942^b^ (− 0.5887 to − 0.1727)	0.0021*
	Linc00938	Unpaired t test	0.6292^a^ (0.2515 to 1.007)	0.0015*
	ATP13A2	Unpaired t test	0.7367^a^ (0.2857 to 1.188)	0.0018*
	LRRK2	Mann–Whitney test	0.4614^b^ (0.06501 to 0.6468)	0.0151*
Controls vs. Early stage PD patients	Hsa-miR-24-3p	Ordinary one-way ANOVA (Tukey’s)	0.7350^a^ (0.3096 to 1.161)	0.0003*
	Hsa-miR-30c-5p	One-way ANOVA (Kruskal–Wallis test)	12.29	0.0409*
	Linc01128	Ordinary one-way ANOVA (Tukey’s)	0.5405^a^ (0.2403 to 0.8406)	0.0002*
	Linc00938	One-way ANOVA (Kruskal–Wallis test)	− 15.37	0.0061*
	ATP13A2	Ordinary one-way ANOVA (Tukey’s)	− 0.5775^a^ (-1.143 to − 0.01197)	0.0443*
	LRRK2	Ordinary one-way ANOVA (Tukey’s)	− 0.5557^a^ (-0.9432 to − 0.1683)	0.0030*
Controls vs. Late stage PD patients	Hsa-miR-24-3p	Ordinary one-way ANOVA (Tukey’s)	0.9559^a^ (0.4145 to 1.497)	0.0002*
	Hsa-miR-30c-5p	One-way ANOVA (Kruskal–Wallis test)	10.70	0.2738
	Linc01128	Ordinary one-way ANOVA (Tukey’s)	0.02435^a^ (− 0.3532 to 0.4019)	0.9868
	Linc00938	One-way ANOVA (Kruskal–Wallis test)	− 18.03	0.0134*
	ATP13A2	Ordinary one-way ANOVA (Tukey’s)	− 1.127^a^ (− 1.847 to − 0.4079)	0.0011*
	LRRK2	Ordinary one-way ANOVA (Tukey’s)	0.01068^a^ (− 0.4823 to 0.5037)	0.9985
Early stage PD patients vs. Late stage PD patients	Hsa-miR-24-3p	Ordinary one-way ANOVA (Tukey’s)	0.2208^a^ (− 0.2950 to 0.7367)	0.5606
	Hsa-miR-30c-5p	One-way ANOVA (Kruskal–Wallis test)	− 1.582	> 0.9999
	Linc01128	Ordinary one-way ANOVA (Tukey’s)	− 0.5161^a^ (− 0.8690 to − 0.1633)	0.0025*
	Linc00938	One-way ANOVA (Kruskal–Wallis test)	− 2.658	> 0.9999
	ATP13A2	Ordinary one-way ANOVA (Tukey’s)	− 0.5499^a^ (− 1.236 to 0.1357)	0.1394
	LRRK2	Ordinary one-way ANOVA (Tukey’s)	0.5664^a^ (0.09662 to 1.036)	0.0144*

^a^CI, was referred to difference among means and ^b^CI, was referred to difference between medians. *The difference between the two groups was significant (P > 0.05).

### Plasmid construction of *Linc00938* and *hsa-miR-30c-5p*

**Figure 5:**
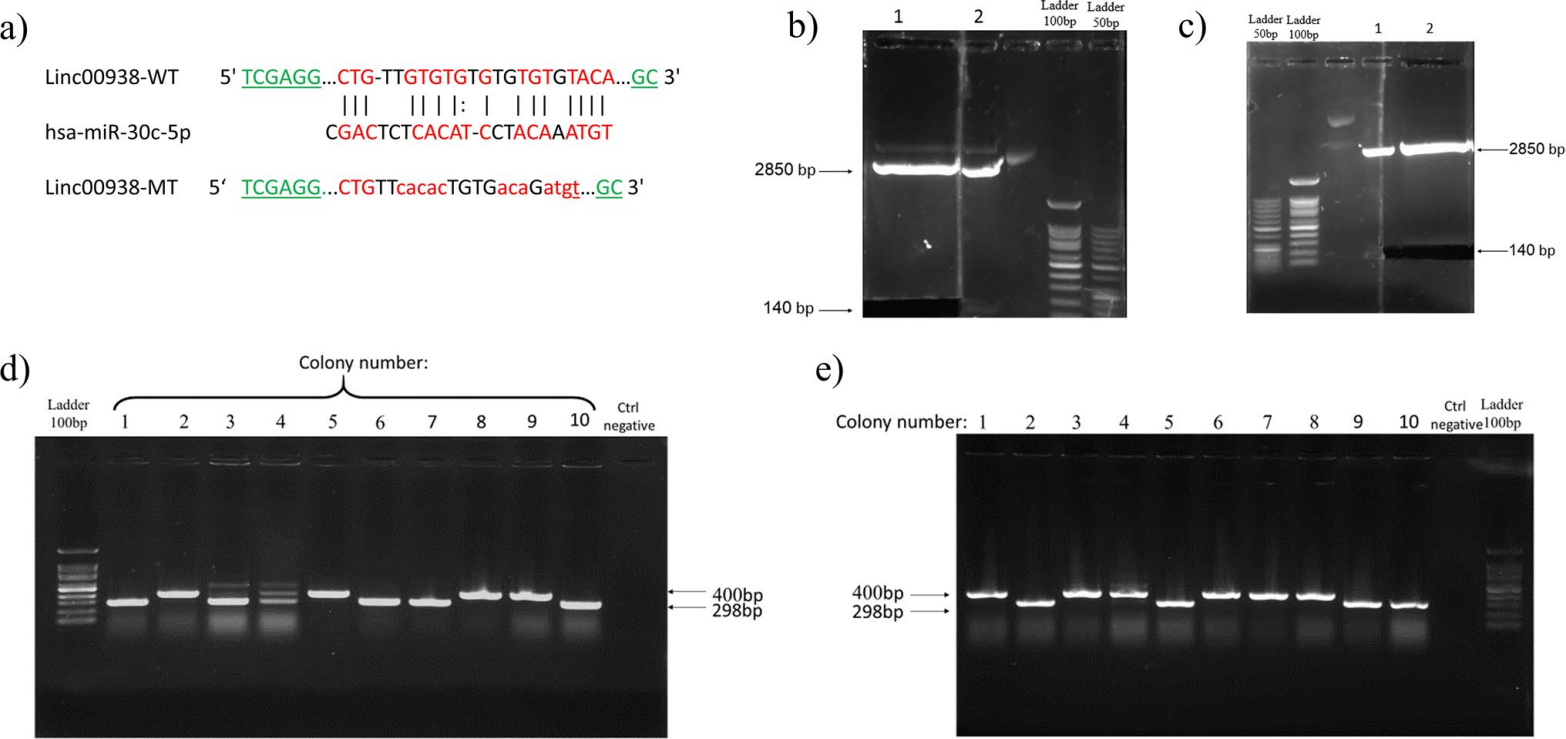
Linc00938 lncRNA regulated LRRK2 expression by directly sponging hsa-miR-30c-5p. (**a**) The predicted binding site between Linc00938 and hsa-miR-30c-5p, and the sequences of wild and mutant types of Linc00938 lncRNA; restriction sites have been underlined. The recombinant plasmids containing mutant and wild-type of linc00938 were termed as “MT” and “WT”, respectively. (**b**, **c**) Gel extraction of purchased Linc00938, which was double digested from the PUC57 vector by agarose gel electrophoresis for: (**b**) Linc00938 mutant, lane 1: fragments of the digested vector, lane2: detector for gel extraction, and (**c**) Linc00938 wild type, lane 1: detector for gel extraction and lane2: fragments of the digested vector. (**d**, **e**) The results of the colony-PCR for psiCHECK-2 recombinant by agarose gel electrophoresis, (**d**) the 2,5,8,9 colonies were the positive colony PCR of the linc00938 mutant, and (**e**) the 1,3,4,6,7,8 colonies were the positive colony PCR of linc00938 wild type. The high resolution figures of the electrophoretic gels are provided as Supplementary Fig. 1.

Double strands of *linc00938* (WT) and (MT) which were inserted in PUC57 plasmid were purchased. Figure 5a shows the seed sequence of *hsa-miR-30c-5p* in *linc00938* (WT) and the altered sequence of *linc00938* (MT), as shown with the lowercase letters. Then, the double digest was implemented using inserted restriction enzyme (*XhoI* and *NotI*); after that, the gel extraction could be used to obtain *linc00938* (WT) and (MT) fragments (140 bp) (Fig. 5b,c). Subsequently, these fragments were cloned in the luciferase reporter psiCHECK-2; we performed the colony PCR using the psiCHECK-2 primers to determine the recombinant plasmid (the details are described in the “Plasmid construction” section). Figure 5d and e show the results of the recombinant psiCHECK-2 colony PCR; the 400 bp band was observed in the colonies with recombinant plasmids (*linc00938* (WT) and (MT)).

**Figure 6:**
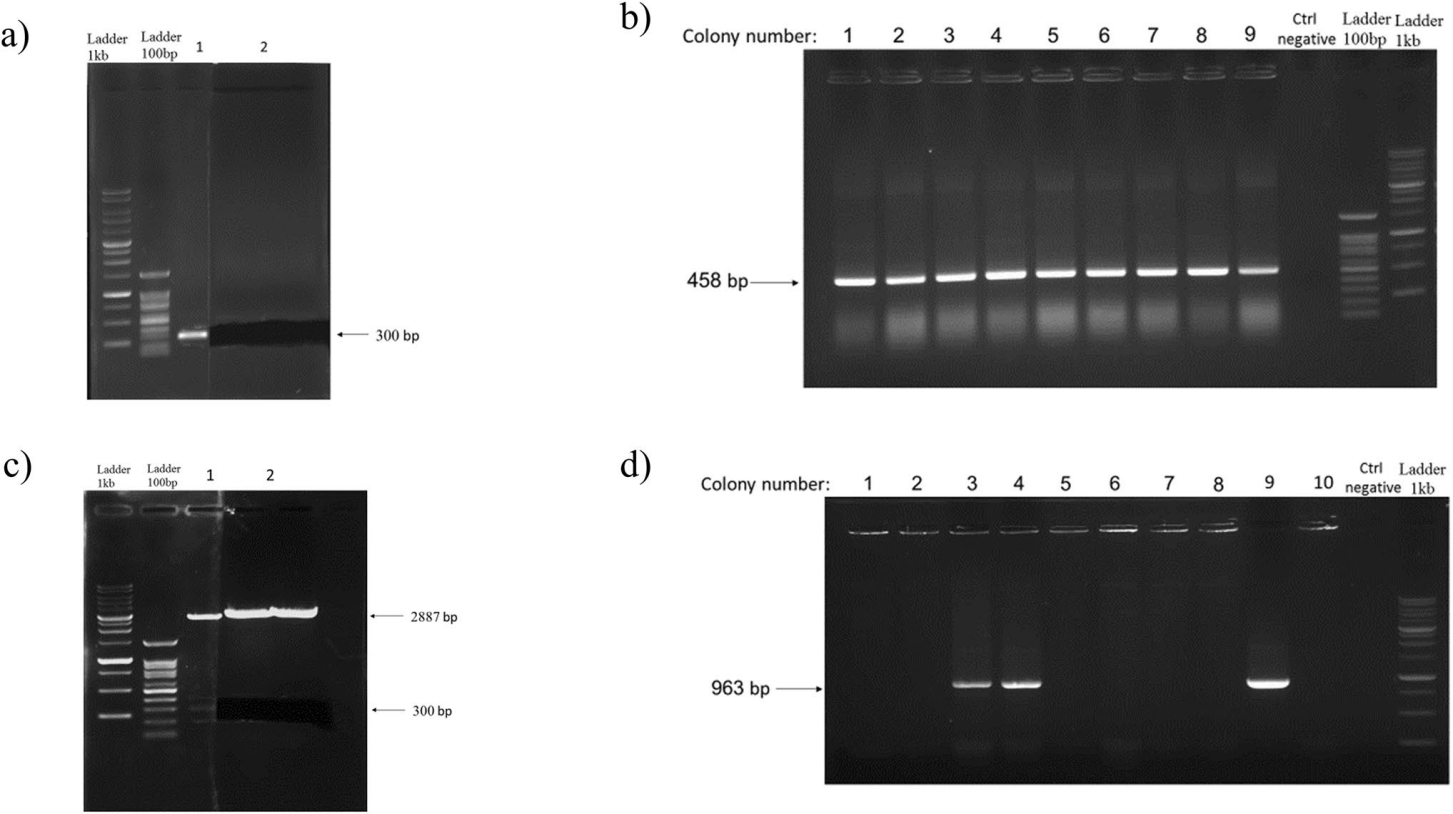
Plasmid construction of hsa-miR-30c. (**a**) Gel extraction of amplified hsa-miR-30c by agarose gel electrophoresis, lane 1: detector for gel extraction and lane2: Gel extraction of hsa-miR-30c PCR fragments. (**b**) PCR colony for the recombinant pTZ57R/T vector, 1 to 9 colonies were positive. (**c**) Gel extraction of hsa-miR-30c, which was double digested from the pTZ57R/T vector by agarose gel electrophoresis, lane 1: detector for gel extraction and lane2: fragments of the digested vector. (**d**) PCR colony for the recombinant pBud-EGFP plasmid, the 3, 4, 9 colonies were the positive colony PCR of the pBud-EGFP plasmid. The high resolution figures of the electrophoretic gels are provided as Supplementary Fig. 2.

The miR-30c precursor was amplified with the designed primers; then, by using the gel extraction, an approximately 300 bp fragment was obtained (Fig. 6a). After ligation of the mir-30c precursor in the pTZ57R/T vector, the recombinant plasmid was determined using the PCR colony with M13 primers (Fig. 6b). Then the positive colonies were selected and double digestion (*HindIII* and *BamHI*) and gel extraction were performed (Fig. 6c). Finally, the miR-30c precursor was cloned into the pBud-EGFP plasmid; the positive colonies were confirmed using colony PCR with CMV forward and miR-30c reverse primers (Fig. 6d) (the details are described in the “Plasmid construction” section).

### *Linc00938* directly sponging the *hsa-miR-30c-5p*

**Figure 7:**
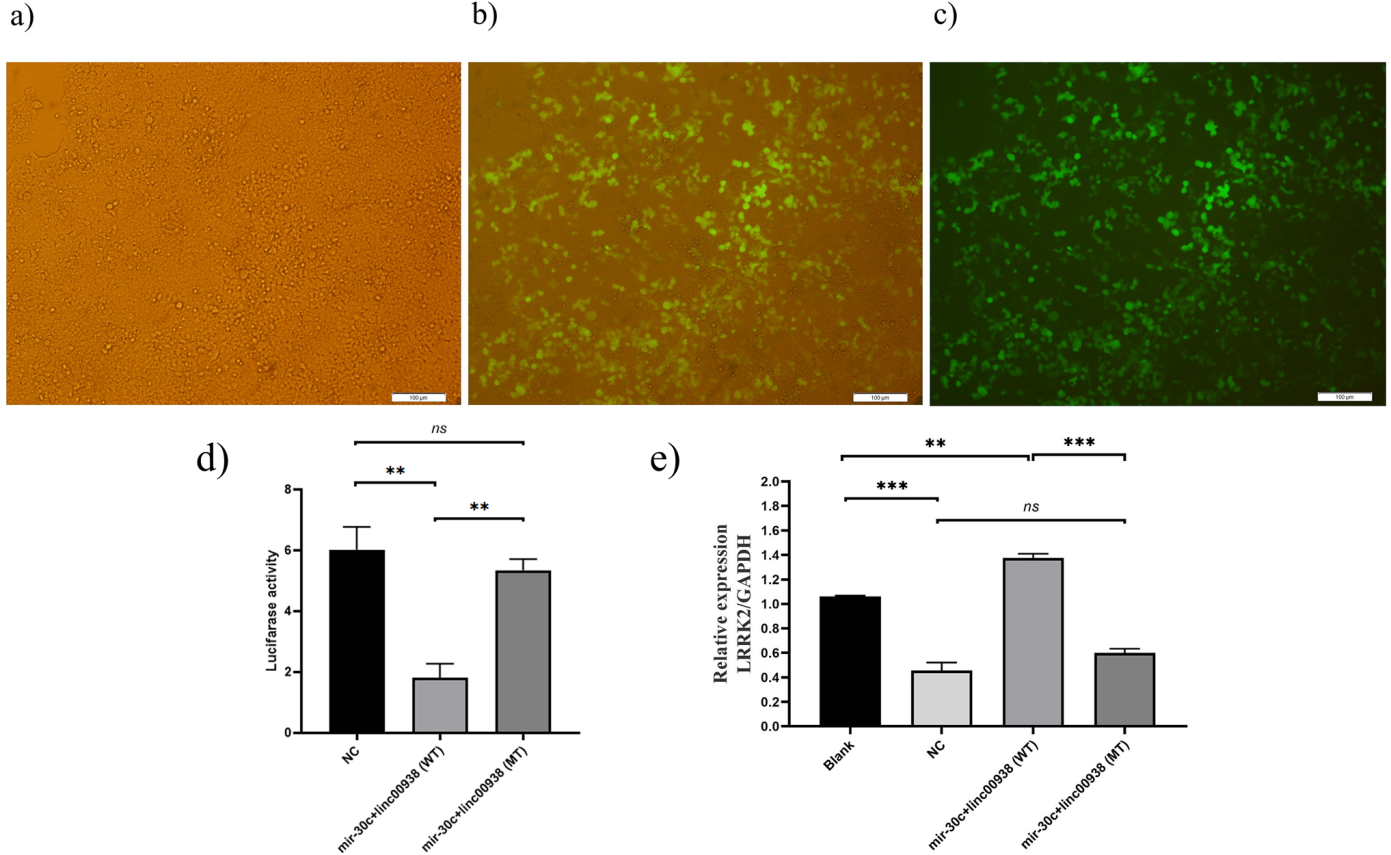
Transfection of constructed plasmids in the HEK293T cells and dual luciferase assay. (**a**–**c**) Fluorescent imaging of HEК293T cells 48 h after transfection using fluorescent microscopy. Magnification × 10. (**d**) The luciferase activity of linc00938-WT, linc00938-MT and psiCHECK (empty vector) in the HEK293T cells treated with hsa-mir-30c. (**e**) Relative mRNA levels of LRRK2 determined by real-time PCR analysis after transfection with psiCHECK-linc00938 (WT) + pBud-mir-30c, psiCHECK-linc00938 (MT) + pBud-mir-30c, NC, and cells without any vector (Blank). Error bars represent SD. (**p ≤ 0.01, and ns p > 0.05).

Transfection of the constructed pBud-mir-30c plasmid was confirmed using a fluorescent microscope; fluorescence images are shown in Fig. 7a–c. The results of the dual luciferase assay showed 70.1% decrease of the luciferase activity in the transfected cells with psiCHECK-linc00938 (WT) + pBud-mir-30c, as compared with the negative control (Fig. 7d); *hsa-miR-30c-5p* significantly inhibited luciferase activity of *linc00938 (WT).* However, *hsa-miR-30c-5p* had no special impact on the luciferase activity of *linc00938 (MT),* when the binding site of hsa-miR-30c was mutated. These results, therefore, demonstrated that *miR-30c* targets *linc00938* by the miR-30c-binding site located in the *linc00938* fragments, thus inhibiting its transcriptional activity. Also, the analysis of the qRT-PCR of *LRRK2* expression in various transfected cells demonstrated the overexpression of *LRRK2* mRNA in the transfected cells with psiCHECK-linc00938 (WT) + pBud-mir-30c, as compared with the Blank, NC and transfected cells with psiCHECK-linc00938 (MT) + pBud-mir-30c (Fig. 7e).

### The regulatory miRNA-based network construction

**Figure 8:**
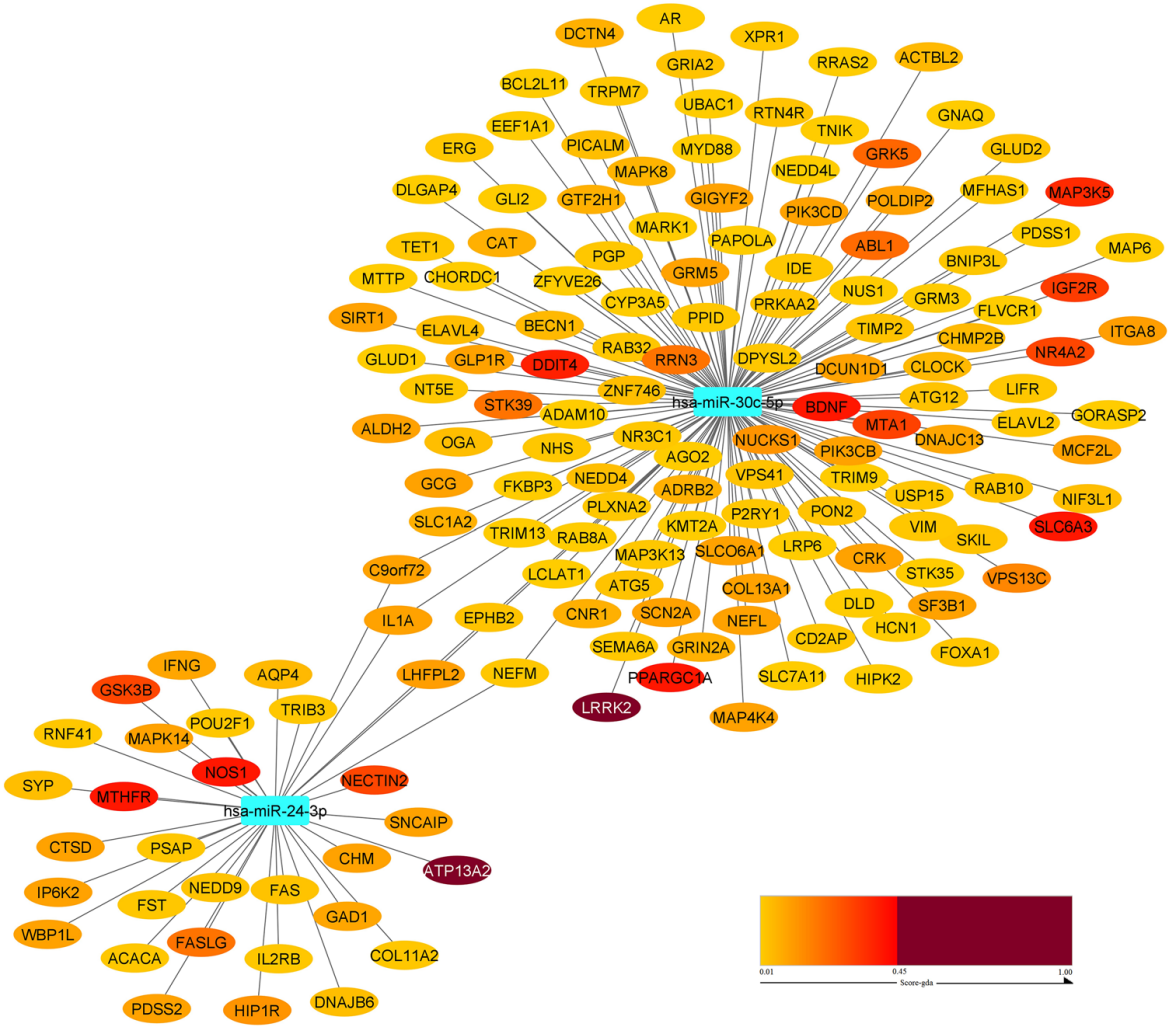
Construction of the regulatory network based on two miRNAs were dysregulated in PD. The common genes are IL1A, LHFPL2, EPHB2, NEFM and C9orf79.

Hsa-miR-30c-5p interacted with 131 PD-related genes (LRRK2 has the highest score among these genes). Also, hsa-miR-24-3p interacted with 34 PD-related genes (ATP13A2 has the highest score among these genes). Figure 8 shows a regulatory network based on two miRNAs constructed; this network consisted of 162 nodes (2 miRNAs, 155 target genes and 5 common target genes). According to the *Enrichr* database^32^, these common genes (*IL1A, LHFPL2, EPHB2*, and *NEFM*) also contributed to several disorders, such as memory impairment, mental depression, depressive disorder, bipolar disorder, and so on.

## Discussion

PD is regarded as the second most popular neurodegenerative disease; the etiology of this disease is not yet known; however, genetic as well as environmental factors seem to play a critical role in causing PD^33^. MiRNAs regulate target gene expression, particularly through post-transcriptional regulation, as well as abnormal miRNA expression involved in the pathogenesis of PD^34^. LncRNAs also have regulatory roles for gene expression through different ways. LncRNAs could do the regulation of the genes expression by altering the expression of miRNAs^6^; they can also bind directly to the DNA sequence and block the transcriptional process^35^.

The miR-30c-5p target gene (*LRRK2*) is involved in various cellular pathways that can lead to PD. *LRRK2* is involved in mitochondrial dysfunction, total level of intracellular ATP, mitochondrial fission, mitochondrial trafficking and oxidative stress^36–38^. Patients carrying the *LRRK2* G2019S homozygous or R1441C heterozygous mutation display the increased damage of mitochondrial DNA (mtDNA)^39^. Another study lacking the *LRRK2* homolog in the *C. elegans* model suggested that *LRRK2* is critical to prevent ER stress and spontaneous neurodegeneration^40^. *LRRK2* regulates anterograde ER-Golgi transport by anchoring Sec16A at ER exit sites, leading to a reduction in the ER stress^41^. *LRRK2* plays a role in converging ER export and secretory trafficking^42^. On the other hand, *LRRK2* is required for the ER exit site assembly and efficient ER cargo exit^41^. Overexpression of wild-type or disease-associated mutations could lead to trans-Golgi-network marker ablation, possibly resulting in the accumulation of the secretory cargo^43^. Knockdown of *LRRK2* increases autophagic activity and reduces cell death^44^. *LRRK2* mutations or knockout of *LRRK2* can result in the reduction of the autophagic marker LC3-II^45,46^. Alteration in the GTPase activity of the *LRRK2* protein impairs endocytic vesicular trafficking and autophagy^47^. *LRRK2* knockout mice exhibited reduced macroautophagy^48^. Knockdown of *LRRK2* (i.e. silencing of its kinase activity) in immune cells resulted in the impairment of macroautophagy and reduction of its degradative capacity^49^. *LRRK2* inhibition decreases normal fission, leading to the elongation of the mitochondrial network which contributes to the poor degradation of the deficient mitochondria^50^. *LRRK2* may, thus, have a regulatory role in the autophagic and endosomal pathways^51^. A dramatic delay of trafficking out of late endosomes was observed in the cells overexpressing G2019S and R1441C *LRRK2*^52^.

Also, the miR-24-3p target gene (*ATP13A2*) is involved in various cellular pathways that can lead to PD. Overexpression of *ATP13A2* can reduce the basal intracellular Ca2+ levels and *ATP13A2* silencing leads to a drop of cytosolic Ca2+ and fragmented mitochondria in cortical neurons^53^. Loss of *ATP13A2* function can affect mitochondrial function, change the levels of ATP, increase fragmentation and production of reactive oxygen species (ROS), and contribute to defective mitophagy^54,55^. *ATP13A2* leads to its retention at the ER, thus triggering the induction of the chronic ER stress and cell death^56^. Lack of *ATP13A2* causes age-related motor dysfunctions and endo-lysosomal dysfunctions, defects independent of α-Syn expression^57^. *ATP13A2* codes for a lysosomal transmembrane protein that functions as P-type ATPase on the lysosome and late endosome^58^. *ATP13A2* deficiency leads to the reduced capacity of lysosomal degradation, resulting in *SNCA* accumulation and neurotoxicity^59^. Silencing of *ATP13A2* induces the fragmentation of mitochondria in a neuronal cell model; its overexpression delays mitochondrial fragmentation. Thus, it plays a role for *ATP13A2* in the quality control of mitochondria, probably through mitophagy^53^.

In this study, we investigated two PD triple networks, the nodes of the first network involving *Linc01128, hsa-miR-24-3p* and *ATP13A2*. The second network nodes were *Linc00938*, *hsa-miR-30c-5p* and *LRRK2*. On the other hand, lncRNAs have different transcripts; so, we obtained the sequence all transcripts of the two selected lncRNAs (*Linc01128* and *Linc00938*); the designed primers that could bind to most transcripts of each lncRNA.

In the experimental results of the first network, we found that the differential expression (DE) of *ATP13A2* were remarkably raised in the PD PBMC, in comparison to the controls. Ramirez et al. also reported the significant overexpression of *ATP13A2* mRNA levels in the post-mortem brain samples of idiopathic PD patients, as compared to controls^60^. In the present study, it was confirmed that the *hsa-miR-24-3p* expression level was significantly downregulated in the PD PBMC, in comparison to the controls; Marques et al. also showed the significant reduction of miR-24 level in cerebrospinal fluid (CSF) of PD, as compared to controls^61^. We also showed the significant decrease of the *Linc01128* expression in the patients with PD; however, the association of *Linc01128* with PD is still unclear in the literature.

The *miRDB* database predicts that the interaction score of *hsa-miR-24-3p* with *ATP13A2* is 88 out of 100 (prediction score above 80 is most likely to be real). As mentioned, this study showed a decrease in the *hsa*-*miR*-*24*-*3p* levels and an increase in the *ATP13A2* levels. On the other hand, hsa-miR-24-3p may bind to *ATP13A2* mRNA, regulating the gene expression. Thus, probably, when the *hsa-miR-24-3p* level was decreased, this regulation was removed and *ATP13A2* expression was increased. *LncRRIsearch* database predicted two interactions between *Linc01128* and *ATP13A2* by RIblast; so, the low expression of *Linc01128* and the high expression of *ATP13A2* could be explained by the fact that lncRNAs play a regulatory role in the target gene by binding directly to the DNA sequence, thus blocking the transcription process. Therefore, when the *Linc01128* level was decreased, this regulation was removed and the *ATP13A2* expression was increased.

Therefore, the increase in the *ATP13A2* expression, as shown in this study, could be justified by two independent methods, one by binding *hsa-miR-24-3p* to *ATP13A2* and the other by directly binding *Linc01128* to *ATP13A2*. Therefore, the *hsa-miR-24-3p* and *Linc01128* levels were decreased in PD; as a result, the expression of *ATP13A2 was* increased in this disease.

In this study, we also investigated DE of the nodes of the second network, including *Linc00938*, *hsa-miR-30c-5p* and *LRRK2*. We examined the LRRK2 mRNA in the PBMC of the PD and healthy individuals. The *LRRK2* level was found to be considerably increased in the PD patients, in comparison to the controls; the *LRRK2* level also showed a significant rise in the early stage patients when compared to controls and late stage groups; as well there was no considerable difference in terms of the *LRRK2* levels in the late stage groups of the PD patients, in comparison to the controls. In 2017, Dzamko et al. also demonstrated that the *LRRK2* level was increased by 30% in substantia nigra of the cases with restricted Lewy bodies; they also reported that *LRRK2* levels had a negative correlation with the disease duration, as compared to controls^62^. This study also demonstrated that the level of *hsa-miR-30c-5p* had a significant decrease in the PD patients PBMC, in comparison to the controls. Similarly, in 2014, Vallelunga et al. reported that miR-30c had downregulation in the serum samples of the PD patients, in comparison to the healthy controls^12^. As well, the expression of *Linc00938* was significantly increased in the PD patients, in comparison to the controls; however, the association of the *Linc00938* with PD is still unclear in the literature.

The miRDB database predicts that the interaction score of *hsa-miR-30c-5p* with *LRRK2* is 93 out of 100; however, LncRRIsearch database does not predict any interaction between *Linc00938* and *LRRK2*. On the other hand, DIANA-LncBase experimental database reported that the interaction score of *hsa-miR-30c-5p* with *Linc00938* was 0.79 out of 1.00, using the immunoprecipitation method^63^; we also demonstrated that *hsa-miR-30c-5p* could directly bind to *Linc00938* by using the luciferase assay. Therefore, the assumption is that the high expression level of *Linc00938* reduces the *hsa-miR-30c-5* expression level by sponging, thus increasing the expression level of *LRRK2*.

In this study, the expression profiles of RNAs were investigated. So, the protein target of hsa-miR-30c-5p requires experimental validation in future investigations.

## Conclusion

The expression profiles of six nodes from two ceRNA networks were examined in this study. For the first time, the association of *Linc01128* and *Linc00938* expression differences with PD was investigated. The results also showed that *Linc00938* could bind directly to hsa-miR-30c-5p, thus possibly regulating *LRRK2* expression via the *miR-30c-5p* sponge. Based on these results, this study could provide new insights into the lncRNA-miRNA-mRNA ceRNA network in the PD gene regulator. In the future studies, *Linc01128* and *Linc00938* could be further considered as PD biomarkers in the diagnosis and treatment of PD.

## Supplementary Information

Supplementary Figure S1.

Supplementary Figure S2.

## References

[1] Eriksen JL, Wszolek Z, Petrucelli L (2005). Molecular pathogenesis of parkinson disease. Arch. Neurol..

[2] Schapira AH (2009). Etiology and pathogenesis of Parkinson disease. Neurol. Clin..

[3] Balestrino R, Schapira A (2020). Parkinson disease. Eur. J. Neurol..

[4] Ayano G (2016). Parkinson's disease: A concise overview of etiology, epidemiology, diagnosis, comorbidity and management. J. Neurol. Disord..

[5] Jonas S, Izaurralde E (2015). Towards a molecular understanding of microRNA-mediated gene silencing. Nat. Rev. Genet..

[6] Chen YG, Satpathy AT, Chang HY (2017). Gene regulation in the immune system by long noncoding RNAs. Nat. Immunol..

[7] Margis R, Margis R, Rieder CRM (2011). Identification of blood microRNAs associated to Parkinsońs disease. J. Biotechnol..

[8] Soreq L (2013). Small RNA sequencing-microarray analyses in Parkinson leukocytes reveal deep brain stimulation-induced splicing changes that classify brain region transcriptomes. Front. Mol. Neurosci..

[9] Boros FA, Maszlag-Török R, Vécsei L, Klivényi P (2020). Increased level of NEAT1 long non-coding RNA is detectable in peripheral blood cells of patients with Parkinson’s disease. Brain Res..

[10] Alieva AK (2015). miRNA expression is highly sensitive to a drug therapy in Parkinson's disease. Parkinsonism Relat. Disord..

[11] Cao X-Y (2017). MicroRNA biomarkers of Parkinson’s disease in serum exosome-like microvesicles. Neurosci. Lett..

[12] Vallelunga A (2014). Identification of circulating microRNAs for the differential diagnosis of Parkinson's disease and Multiple System Atrophy. Front. Cell. Neurosci..

[13] Huang H-Y (2019). miRTarBase 2020: Updates to the experimentally validated microRNA–target interaction database. Nucleic Acids Res..

[14] Paraskevopoulou MD (2013). DIANA-LncBase: Experimentally verified and computationally predicted microRNA targets on long non-coding RNAs. Nucleic Acids Res..

[15] Kanehisa M, Furumichi M, Tanabe M, Sato Y, Morishima K (2017). KEGG: New perspectives on genomes, pathways, diseases and drugs. Nucleic Acids Res..

[16] Piñero J (2019). The DisGeNET knowledge platform for disease genomics: 2019 update. Nucleic Acids Res..

[17] Bao Z (2019). LncRNADisease 2.0: An updated database of long non-coding RNA-associated diseases. Nucleic Acids Res..

[18] Volders P-J (2019). LNCipedia 5: Towards a reference set of human long non-coding RNAs. Nucleic Acids Res..

[19] Fang S (2018). NONCODEV5: A comprehensive annotation database for long non-coding RNAs. Nucleic Acids Res..

[20] Chen Y, Wang X (2020). miRDB: An online database for prediction of functional microRNA targets. Nucleic Acids Res..

[21] Fukunaga T, Iwakiri J, Ono Y, Hamada M (2019). LncRRIsearch: A web server for lncRNA-RNA interaction prediction integrated with tissue-specific expression and subcellular localization data. Front. Genet..

[22] Kohl M, Wiese S, Warscheid B (2011). Data Mining in Proteomics.

[23] Kozomara A, Birgaoanu M, Griffiths-Jones S (2018). miRBase: From microRNA sequences to function. Nucleic Acids Res..

[24] Miranda KC (2006). A pattern-based method for the identification of MicroRNA binding sites and their corresponding heteroduplexes. Cell.

[25] Cooper, P., Landrum, M., Mizrachi, I. & Weisemann, J. *Entrez Sequences Quick Start*. (National Center for Biotechnology Information, 2016).

[26] Rehmsmeier M, Steffen P, Höchsmann M, Giegerich R (2004). Fast and effective prediction of microRNA/target duplexes. RNA.

[27] Poewe W (2017). Parkinson disease. Nat. Rev. Dis. Primers.

[28] Hughes AJ, Daniel SE, Kilford L, Lees AJ (1992). Accuracy of clinical diagnosis of idiopathic Parkinson's disease: A clinico-pathological study of 100 cases. J. Neurol. Neurosurg. Psychiatry.

[29] Fahn, S., Elton, R. & Committee, U. D. (2015).

[30] Nasreddine ZS (2005). The Montreal Cognitive Assessment, MoCA: A brief screening tool for mild cognitive impairment. J. Am. Geriatr. Soc..

[31] Jodoin J (2009). Loss of anti-Bax function in Gerstmann-Sträussler-Scheinker syndrome-associated prion protein mutants. PLoS ONE.

[32] Kuleshov MV (2016). Enrichr: A comprehensive gene set enrichment analysis web server 2016 update. Nucleic Acids Res..

[33] Caggiu E (2018). Differential expression of miRNA 155 and miRNA 146a in Parkinson's disease patients. ENeurol. Sci..

[34] Ma L (2013). Advances with microRNAs in Parkinson’s disease research. Drug Des. Dev. Ther..

[35] Wu Z (2014). Regulation of lncRNA expression. Cell. Mol. Biol. Lett..

[36] Sai Y, Zou Z, Peng K, Dong Z (2012). The Parkinson's disease-related genes act in mitochondrial homeostasis. Neurosci. Biobehav. Rev..

[37] Wang X (2012). LRRK2 regulates mitochondrial dynamics and function through direct interaction with DLP1. Hum. Mol. Genet..

[38] Park J-S, Davis RL, Sue CM (2018). Mitochondrial dysfunction in Parkinson’s disease: New mechanistic insights and therapeutic perspectives. Curr. Neurol. Neurosci. Rep..

[39] Sanders LH (2014). Mitochondrial DNA damage: Molecular marker of vulnerable nigral neurons in Parkinson's disease. Neurobiol. Dis..

[40] Sämann J (2009). *Caenorhabditits elegans* LRK-1 and PINK-1 act antagonistically in stress response and neurite outgrowth. J. Biol. Chem..

[41] Cho HJ (2014). Leucine-rich repeat kinase 2 regulates Sec16A at ER exit sites to allow ER–Golgi export. EMBO J..

[42] Wang T, Hay JC (2015). Alpha-synuclein toxicity in the early secretory pathway: How it drives neurodegeneration in Parkinsons disease. Front. Neurosci..

[43] Beilina A (2014). Unbiased screen for interactors of leucine-rich repeat kinase 2 supports a common pathway for sporadic and familial Parkinson disease. Proc. Natl. Acad. Sci..

[44] Alegre-Abarrategui J (2009). LRRK2 regulates autophagic activity and localizes to specific membrane microdomains in a novel human genomic reporter cellular model. Hum. Mol. Genet..

[45] Hakimi M (2011). Parkinson’s disease-linked LRRK2 is expressed in circulating and tissue immune cells and upregulated following recognition of microbial structures. J. Neural Transm..

[46] Tong Y (2010). Loss of leucine-rich repeat kinase 2 causes impairment of protein degradation pathways, accumulation of α-synuclein, and apoptotic cell death in aged mice. Proc. Natl. Acad. Sci..

[47] Xiong Y (2010). GTPase activity plays a key role in the pathobiology of LRRK2. PLoS Genet..

[48] Orenstein SJ (2013). Interplay of LRRK2 with chaperone-mediated autophagy. Nat. Neurosci..

[49] Schapansky J, Nardozzi JD, Felizia F, LaVoie MJ (2014). Membrane recruitment of endogenous LRRK2 precedes its potent regulation of autophagy. Hum. Mol. Genet..

[50] Esteves A, G-fernandes M, Santos D, Januário C, Cardoso S (2015). The upshot of LRRK2 inhibition to Parkinson’s disease paradigm. Mol. Neurobiol..

[51] Roosen DA, Cookson MR (2016). LRRK2 at the interface of autophagosomes, endosomes and lysosomes. Mol. Neurodegener..

[52] Gómez-Suaga P (2014). LRRK2 delays degradative receptor trafficking by impeding late endosomal budding through decreasing Rab7 activity. Hum. Mol. Genet..

[53] Ramonet D (2012). PARK9-associated ATP13A2 localizes to intracellular acidic vesicles and regulates cation homeostasis and neuronal integrity. Hum. Mol. Genet..

[54] Gusdon AM, Zhu J, Van Houten B, Chu CT (2012). ATP13A2 regulates mitochondrial bioenergetics through macroautophagy. Neurobiol. Dis..

[55] Park JS, Blair NF, Sue CM (2015). The role of ATP13A2 in Parkinson's disease: Clinical phenotypes and molecular mechanisms. Mov. Disord..

[56] Park JS (2011). Pathogenic effects of novel mutations in the P-type ATPase ATP13A2 (PARK9) causing Kufor-Rakeb syndrome, a form of early-onset parkinsonism. Hum. Mutat..

[57] Kett LR (2015). α-Synuclein-independent histopathological and motor deficits in mice lacking the endolysosomal Parkinsonism protein Atp13a2. J. Neurosci..

[58] da Fonseca TL, Outeiro TF (2014). ATP13A2 and alpha-synuclein: A metal taste in autophagy. Exp. Neurobiol..

[59] Usenovic M, Tresse E, Mazzulli JR, Taylor JP, Krainc D (2012). Deficiency of ATP13A2 leads to lysosomal dysfunction, α-synuclein accumulation, and neurotoxicity. J. Neurosci..

[60] Ramirez A (2006). Hereditary parkinsonism with dementia is caused by mutations in ATP13A2, encoding a lysosomal type 5 P-type ATPase. Nat. Genet..

[61] Marques TM (2017). MicroRNAs in cerebrospinal fluid as potential biomarkers for parkinson’s disease and multiple system atrophy. Mol. Neurobiol..

[62] Dzamko N (2017). LRRK2 levels and phosphorylation in Parkinson's disease brain and cases with restricted Lewy bodies. Mov. Disord..

[63] Boudreau RL (2014). Transcriptome-wide discovery of microRNA binding sites in human brain. Neuron.

