# Supplementary figures and images for: Significant modulations of linc001128 and linc0938 with miR-24-3p and miR-30c-5p in Parkinson disease
Source: Sci Rep. 2022 Feb 16;12:2569. doi: 10.1038/s41598-022-06539-3 (PMC8850599; doi:10.1038/s41598-022-06539-3)

# Supplementary Fig.1

b)

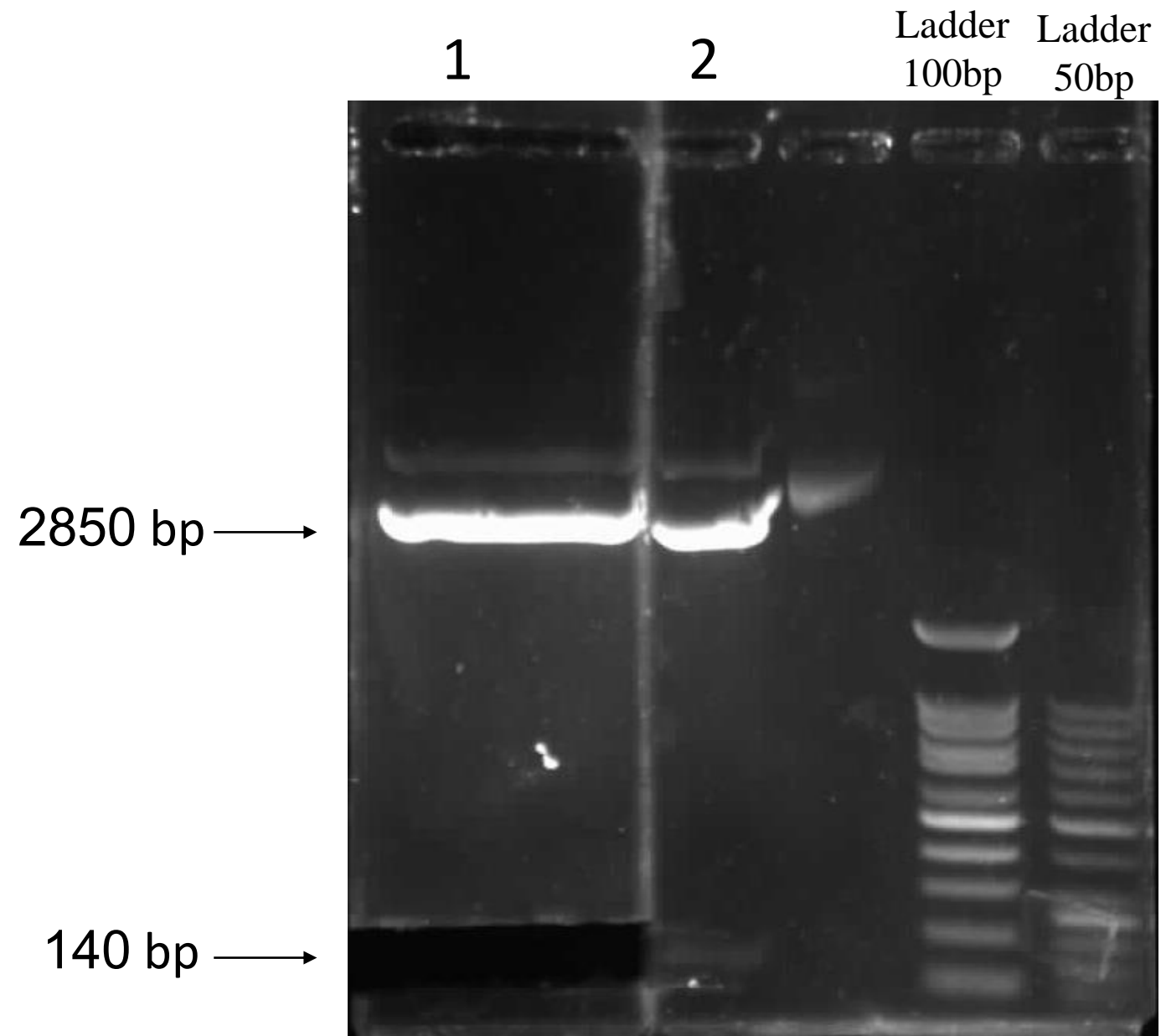

c)

Ladder 50bp    Ladder 100bp

1

2

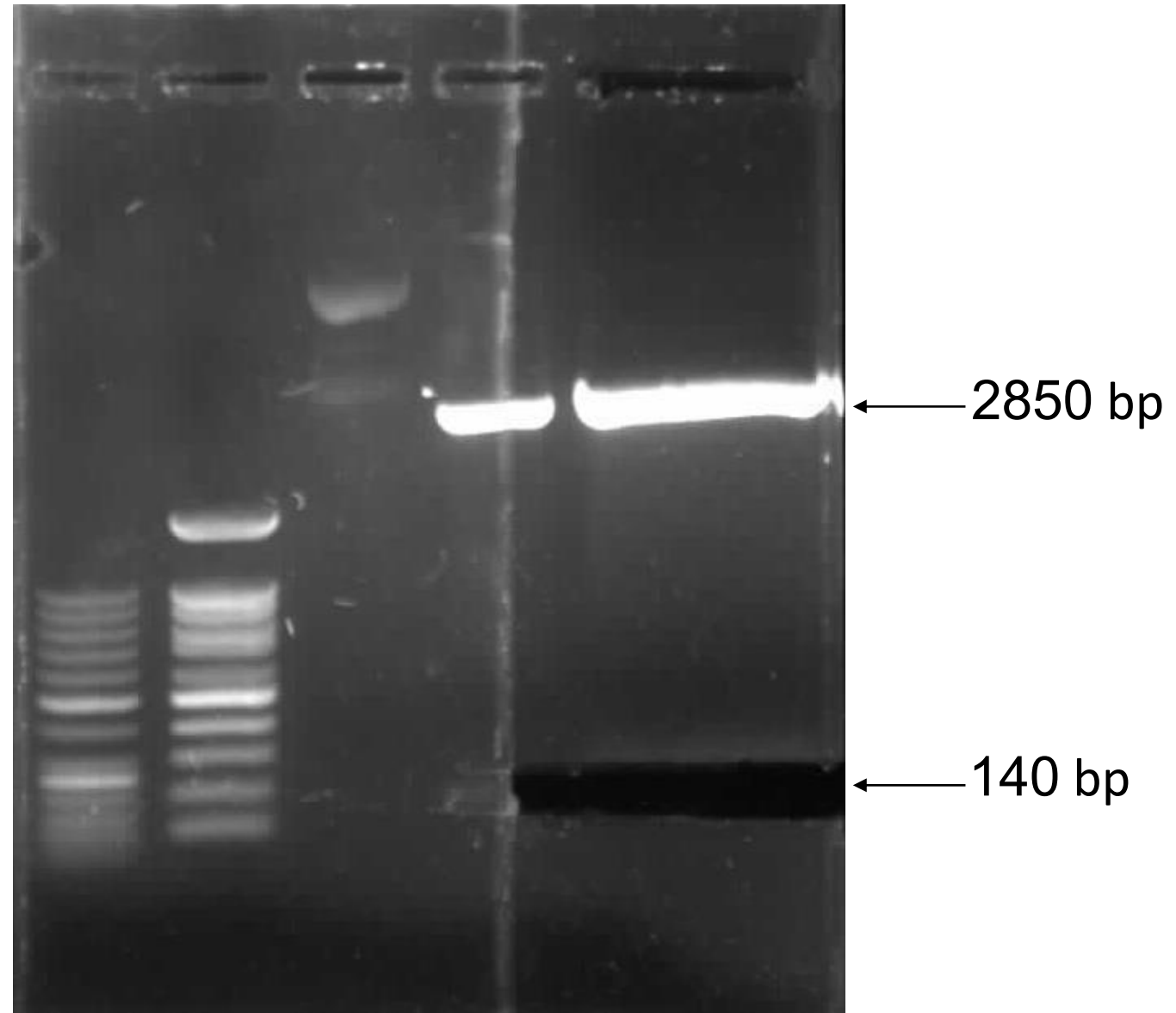

d)

Colony number:

Ladder  
100bp

1

2

3

4

5

6

7

8

9

10

Ctrl  
negative

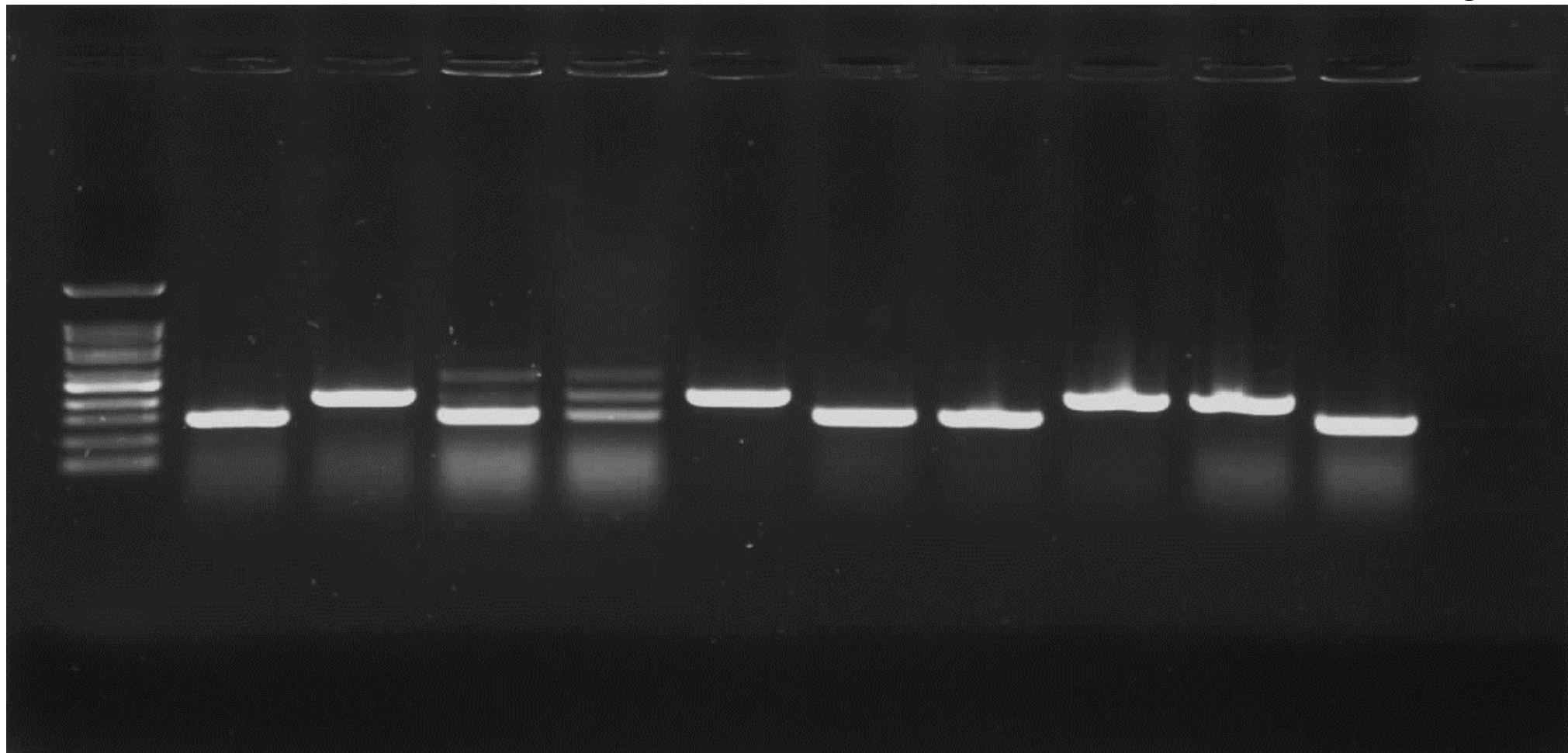

← 400bp

← 298bp

e)

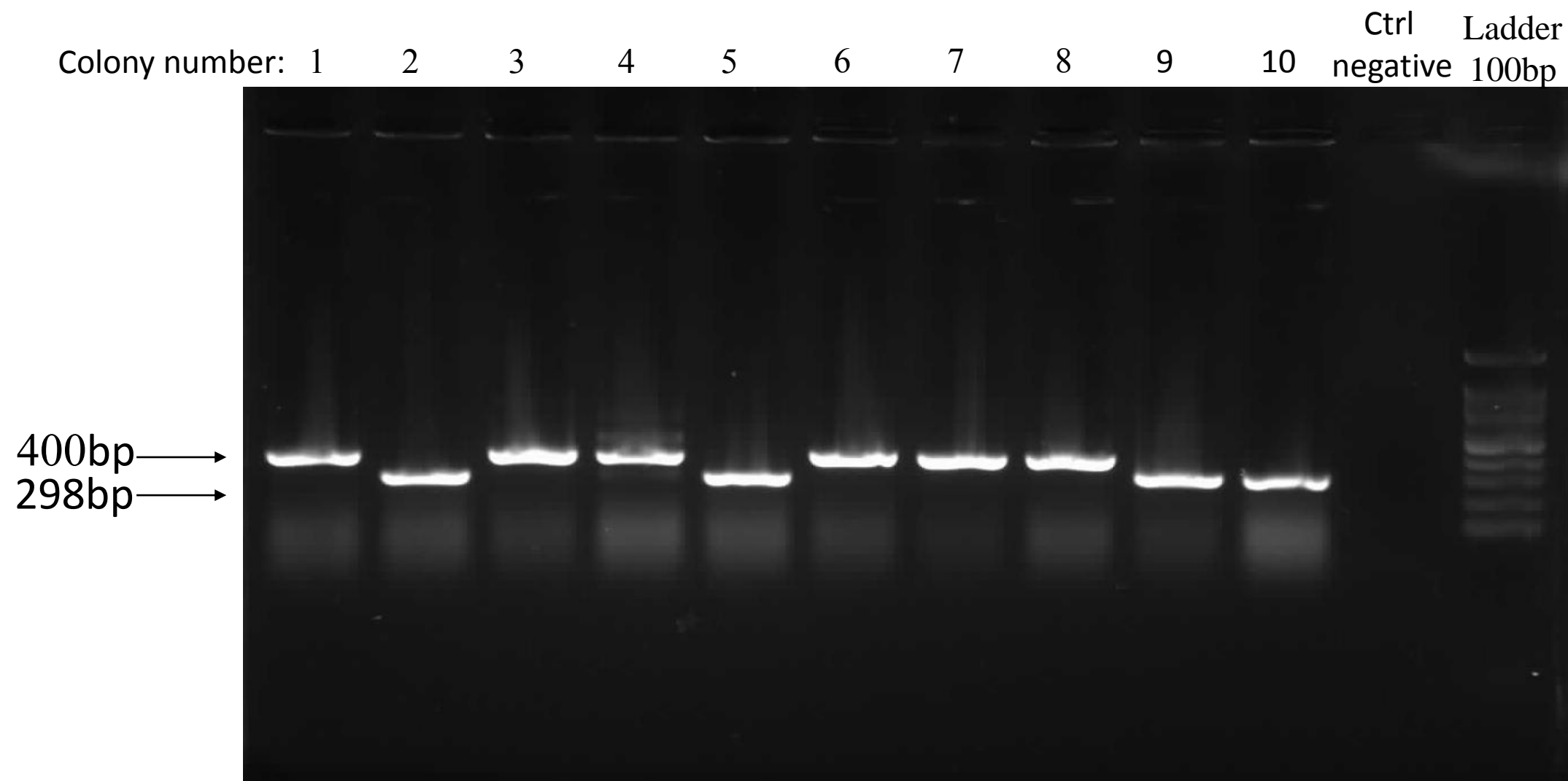

Supplement: Supplementary file 1 — Supplementary Figure S1. [file 41598_2022_6539_MOESM1_ESM.pdf]

# Supplementary Fig.2

a)

Ladder Ladder  
1kb 100bp

1

2

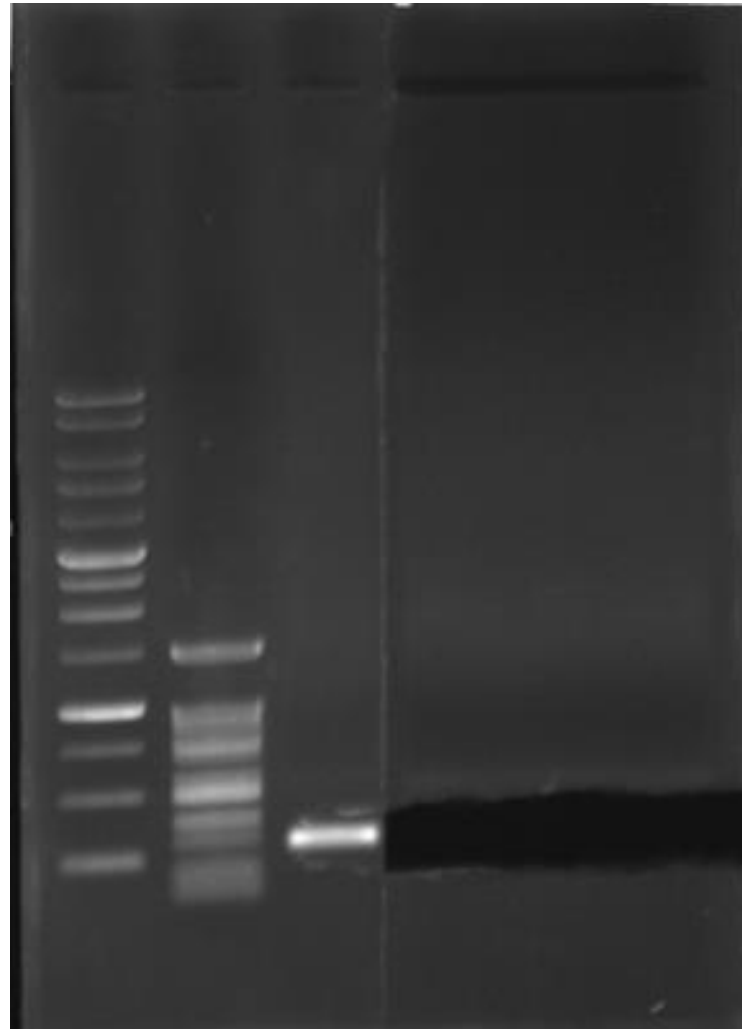

← 300 bp

b)

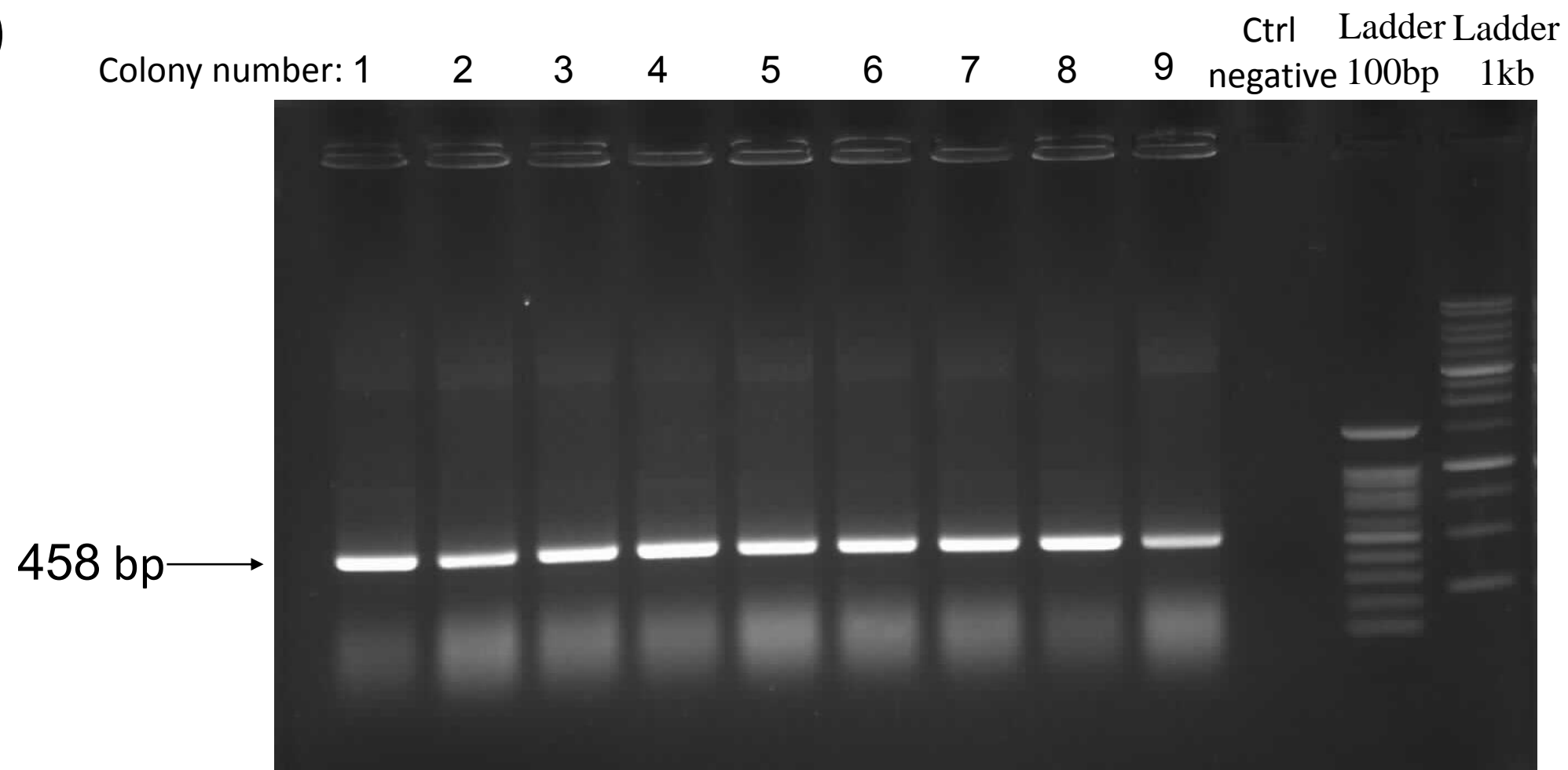

c)

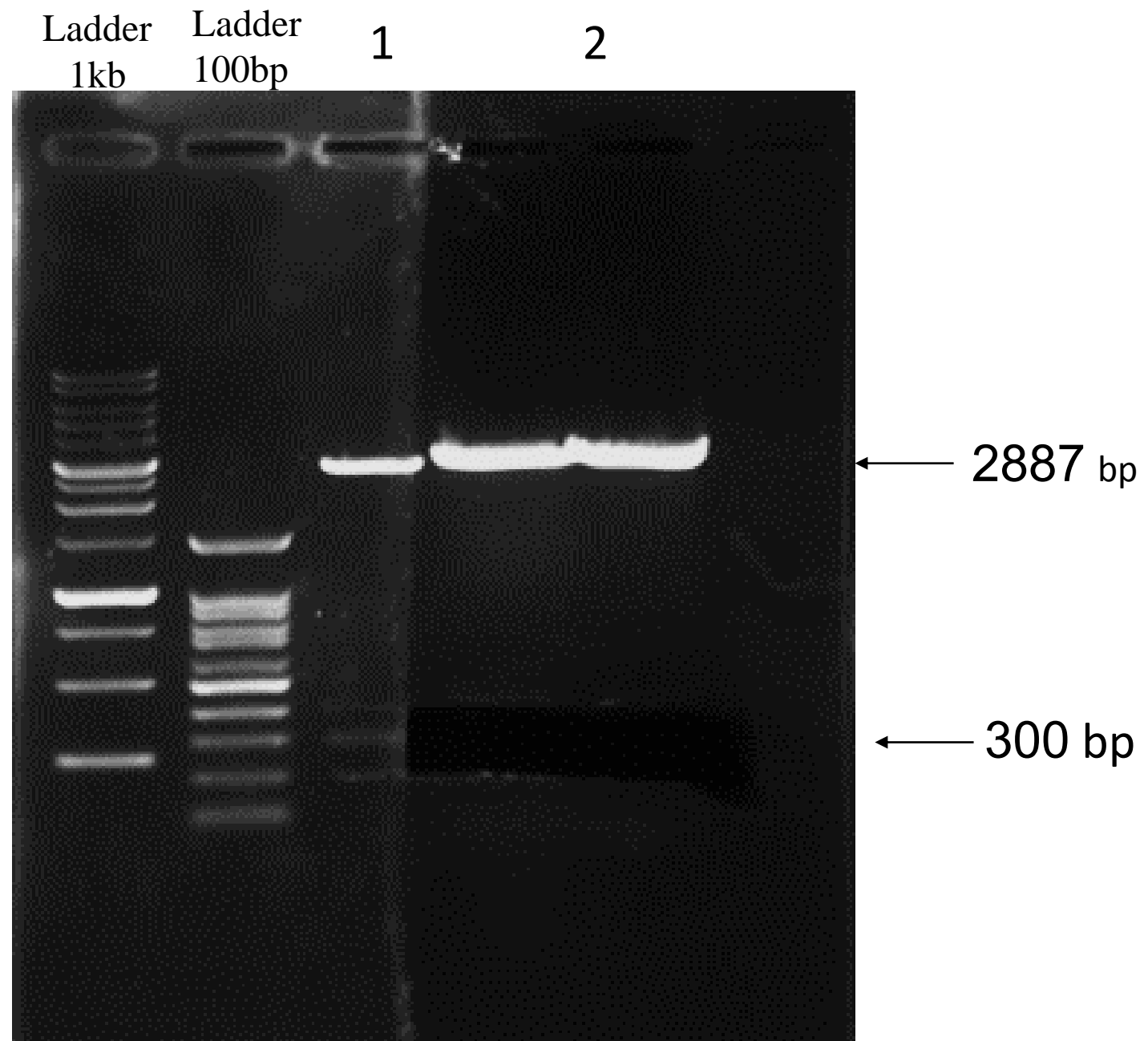

d)

| Colony number: | 1 | 2 | 3 | 4 | 5 | 6 | 7 | 8 | 9 | 10 | Ctrl negative | Ladder 1kb |
|----------------|---|---|---|---|---|---|---|---|---|----|---------------|------------|
|----------------|---|---|---|---|---|---|---|---|---|----|---------------|------------|

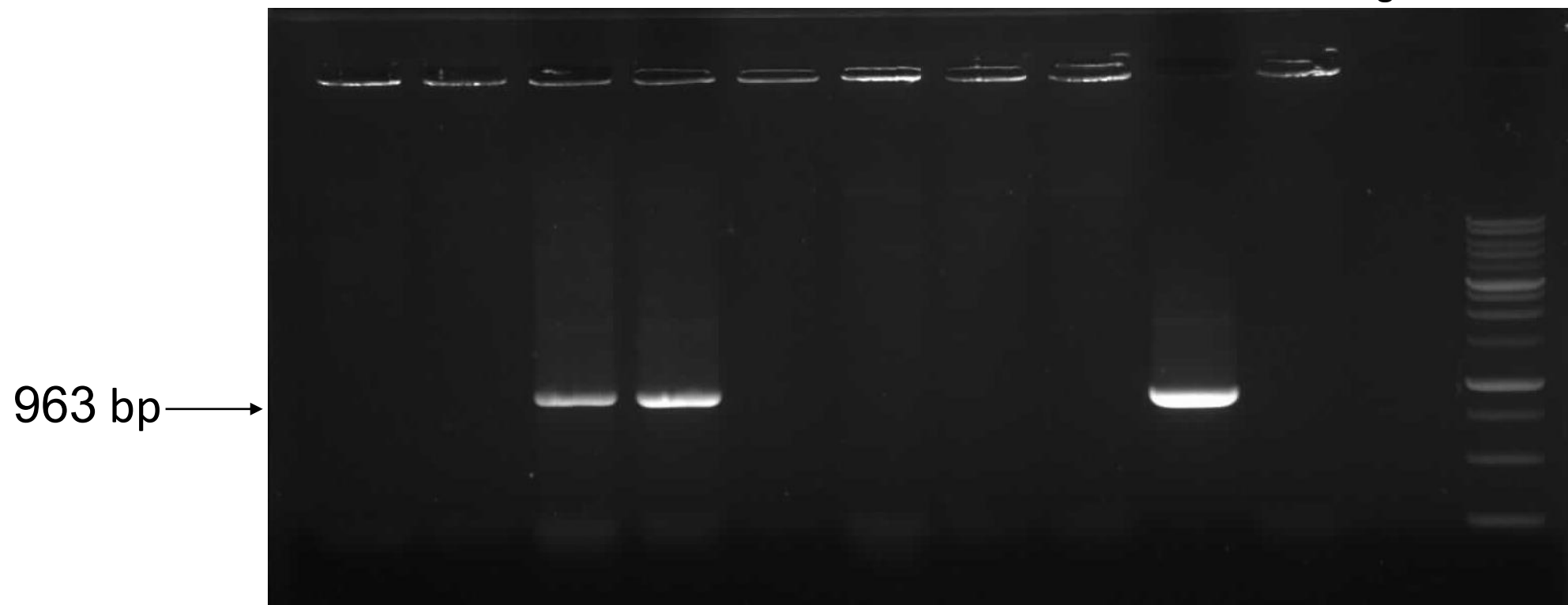

Supplement: Supplementary file 2 — Supplementary Figure S2. [file 41598_2022_6539_MOESM2_ESM.pdf]
